# Spectral efficiency enhancement in multiuser NOMA over Nakagami-*m* fading channels through power allocation and pairing strategies under hardware and SIC imperfections

**DOI:** 10.1038/s41598-025-22024-z

**Published:** 2025-10-31

**Authors:** R Dipinkrishnan, Vinoth Babu Kumaravelu

**Affiliations:** Department of Communication Engineering, School of Electronics Engineering, Vellore Institute of Technology, Vellore, Tamil Nadu 632014 India

**Keywords:** Hardware impairment (HI), Imperfect successive interference cancellation (*i*SIC), Nakagami-*m* fading, Non-orthogonal multiple access (NOMA), Power allocation (PA), User pairing, Engineering, Mathematics and computing

## Abstract

Non-orthogonal multiple access (NOMA) is a key enabler for future sixth-generation (6G) and massive machine-type communication networks, offering superior sum spectral efficiency (SSE) and user fairness compared to traditional orthogonal multiple access (OMA) schemes. However, practical deployments face challenges due to hardware impairments (HI) and imperfect successive interference cancellation (*i*SIC), which cause signal distortion and residual interference, degrading system reliability. Most existing works overlook these impairments or assume ideal fading scenarios, thereby limiting their real-world applicability. This work addresses these issues by developing a comprehensive outage probability analysis for both downlink (DL) and uplink (UL)-NOMA over Nakagami-*m* fading channels, a versatile model for realistic fading conditions. Closed-form expressions are derived using Gamma-function-based modeling, and a low-complexity optimization of power allocation (PA) and user pairing is proposed to enhance system performance under impairments. The results demonstrate that employing a strong–weak (SW) user pairing strategy, along with optimally tuned PA factors, yields a notable improvement in SSE $\approx 15.58\%$ in DL and $\approx 25.55\%$ in UL. The accuracy of the proposed analytical models are substantiated through a strong agreement with simulation outcomes. The integration of user pairing with optimized PA not only enhances spectral efficiency (SE) but also facilitates efficient resource utilization while maintaining low computational complexity. This comprehensive approach positions the proposed system as a viable solution for meeting the performance and scalability requirements of next-generation wireless networks.

## Introduction

The exponential rise in the number of network-connected devices will significantly increase data traffic in future wireless systems. The required quality of service (QoS) demand for the massive number of devices is one of the challenges for the future network. By 2030, it is anticipated that there will be at least 125 billion devices linked to global networks, resulting in a monthly data traffic of 5016 exabytes and a device density of about $10^8$ devices for every square kilometer. In order to accommodate these needs, future networks are expected to achieve a spectral efficiency (SE) of 100 bps/Hz and an energy efficiency (EE) that is 100 times greater than that of fifth-generation (5G)^1^. Non-orthogonal multiple access (NOMA) is a key technology that supports connectivity requirements through efficient spectrum utilization. Unlike orthogonal multiple access (OMA) technology, NOMA allocates power resources among users based on channel conditions^2^. In NOMA, a weighted sum of signals from different users is transmitted regardless of time and frequency. The weights correspond to the power allocation (PA) factors for each user. To decode the data in a NOMA-based network successive interference cancellation (SIC) is used.

The sum spectral efficiency (SSE) and the probability of outages are two key metrics used to evaluate the efficiency of a wireless system. The SSE reflects the overall integrity of the system, whereas the outage probability provides insights into the fairness experienced by individual users^3^. While NOMA offers improved SSE compared to OMA, an increase in the number of connected devices leads to a gradual decline in SSE. This can be resolved by optimizing the PA factor. In NOMA, PA is performed according to the user channel condition. The user located near the gNodeBs (gNB) is assigned a lower PA, and the user far from the gNB is assigned a higher PA. The fixed values of PA factors are inadequate for effectively navigating the varying channel conditions. An efficient PA strategy enhances the SSE of the network while minimizing user outages^4^. This work addresses the SSE enhancement in Nakagami-*m*-based uplink (UL) and downlink (DL) NOMA systems using optimized PA factors. It also derives the closed-form expressions for the probability of outage under different fading conditions.

In NOMA, as the number of users increases, the complexity of SIC increases. The imperfect SIC (*i*SIC) introduces residual information, which leads to error propagation^5^. This arises due to imperfections in the process of sequentially decoding and removing the other user’s data from the received signal. Hardware impairment (HI) is considered another issue related to the wireless system, which arises due to the real-time non-idealities and imperfections of hardware present in the NOMA system^6^. In a real-time scenario, both *i*SIC and HI are unavoidable. NOMA greatly outperforms OMA in environments with varied user channel conditions due to its ability for power-domain multiplexing, which improves SE and provides fair user access. Nevertheless, as the number of users increases, determining the optimal PA becomes increasingly complex, especially when accounting for real-world impairments such as *i*SIC and HI. These adverse effects can be minimized through the implementation of effective user pairing strategies. User pairing can minimize the propagation error due to *i*SIC and can also leverage the advantage of the NOMA system by pairing users with a maximum distance between them^7,8^. This work includes the performance analysis of different user pairing strategies and also compares the average probability of outage of users in a multi-user environment.

This work focuses on the Nakagami-*m* channel model, which is a generalized model suitable to describe the varying channel environment of wireless systems^9^. It can be used to effectively model different fading conditions by adjusting the shape parameter, denoted as *m*. The severe fading scenario corresponds to $m<1$. The Rayleigh fading model is represented by the condition $m=1$. The condition $m>1$ represents the presence of a strong line of sight (LOS) component, using which Rician fading conditions can be modeled. The relation between Rician factor (*K*) and Nakagami-*m* shape parameter (*m*) can be expressed as $m = \frac{{(K+1)}^2}{(2K+1)}$, where $m>1$^10^. For very large *m*, the model approaches a Gaussian channel behavior, resembling a deterministic channel model. Hence, using the Nakagami-*m* model, the analysis of various real-time scenarios, including indoor, outdoor, urban, and suburban environments, can be performed.

### Major contributions

While many studies examine idealized NOMA configurations, comprehensive assessments of both DL and UL-hybrid NOMA (HNOMA) systems under realistic channel conditions and impairments are limited. This research addresses this gap by:

- Developing a comprehensive outage analysis framework for DL and UL-NOMA under Nakagami-*m* fading, which encompasses various wireless environments including Rayleigh and Rician channels.
- Incorporating the effects of HI and *i*SIC into the system model to obtain new closed-form formulas for outage probability and SSE.
- Proposing low-complexity, user-specific PA strategies that adapt to channel statistics to improve SE and fairness.
- Exploring various user pairing methods within multi-user NOMA, demonstrating that pairing strong and weak users combined with optimized PA can significantly boost SSE in both UL and DL.
- Validating the theoretical derivations through simulation results, confirming the analytical accuracy, and reinforcing the practical applicability of the proposed models under realistic system conditions.

### Organization

The manuscript is organized into several sections. Section 1 explores the potential of NOMA for future wireless systems, highlighting the limitations of OMA, the challenges in supporting massive connectivity, and the significance of realistic modeling with Nakagami-*m* fading. Section 2 reviews existing research on outage probabilities, PA, user pairing, and the effects of HI and *i*SIC. Section 3 describes the system models for both DL and UL-NOMA, incorporating the impacts of HI and *i*SIC. Section 4 provides the mathematical derivation of outage probabilities, including *i*SIC and HI conditions. Section 5 presents the SSE achieved by different user pairing strategies in multi-user NOMA systems, while Sect. 6 focuses on enhancing SSE through the optimization of PA factors. Section 7 presents simulation results that verify the accuracy of the analytical expressions and compare different pairing and PA strategies. Finally, Sect. 8 summarizes key findings and suggests directions for future work.

## Related works

This section presents a comprehensive review of existing literature related to NOMA systems, focusing on outage analysis, PA strategies, user pairing techniques, HI, and *i*SIC. It emphasizes the limitations of current research and points out the gaps that this study aims to address.

### Outage probability analysis in NOMA systems

An outage analysis was conducted for multi-user UL and DL-NOMA systems^11^. A generalized framework for fading channels was established by incorporating the Gamma distribution (GD) function. Using this GD function, the probability density function (pdf) and cumulative distribution function (CDF) for a generalized channel model were derived. The accuracy of the theoretical outage analysis was validated through simulations and using the Monte Carlo method. While the study presented a robust theoretical model, it relied on fixed PA factors, which might have limited the performance of the SSE. The Rician channel-based DL-NOMA system and corresponding outage analysis were investigated^12^. HI was addressed in the study to align with real-time scenarios. The suggested study did not incorporate a framework for theoretical modeling. The performance of a DL-NOMA network under both Rician and Rayleigh channel models was examined^13^ . The research provided a closed-form solution for calculating the outage probability. The findings indicated that the Rician model yielded a lower outage probability compared to the Rayleigh model, primarily due to the presence of a strong LOS component. A detailed outage probability derivation for DL-NOMA under Rayleigh fading channel was provided^4^. The suggested work did not consider imperfections in the system.

### PA optimization strategies

The optimization of the PA factor in the DL and UL-NOMA system to enhance the SSE and throughput under *i*SIC was discussed^14^. They incorporated user fairness and power budget as constraints in the optimization framework. Additionally, a user grouping strategy was employed to enhance overall system performance. However, it was noteworthy that the analysis did not address outage probabilities. User fairness could be ensured by minimizing the outage. The PA optimization strategy aimed at reducing outages while maximizing the SSE was addressed^15^. The results compared the performance of OMA and NOMA schemes. The study also evaluated the performance of the optimized PA against fixed PA factors to ensure its effectiveness. The PA optimization in a multi-user DL-NOMA system was investigated^16^. The focus of the suggested study was to design an energy-efficient system with power budget constraints. Intra-cell interference was considered in the analysis to align with the real-time scenario. The Rayleigh fading channel was considered for analysis, and the results were compared with those of the orthogonal frequency division multiple access (OFDMA) scheme. The authors also prioritized minimizing complexity during the optimization process.

The PA optimization approach to maximize the EE was suggested^17^ . The study focused on the minimum fairness demand of the weak user. Average SE and outage were considered as the metrics to analyze the system’s performance. The enhancement of SSE using the optimized PA factors was ensured by comparing it with the performance of fixed PA factors. Although the results indicated improved performance, the approach lacked a solid theoretical foundation. An iterative optimization approach in the DL-NOMA system under the Rician fading channel for cell-free massive multiple-input multiple-output (mMIMO) network was addressed^18^. The importance of pilot signals was discussed in the study through SE and EE analysis. The Rician channel model highlighted the impact of the dominant LOS component.

### User pairing techniques for performance enhancement

User pairing could be used to enhance the SSE in the NOMA system. Employment of user pairing strategies in a multi-user environment with fixed PA factors was discussed^19^. The required QoS level for each user, in terms of SE was considered^20^. The suggested work employed user pairing in fixed NOMA and cognitive radio-inspired NOMA for the analysis. The outage probability and ergodic sum rate were the parameters considered to analyze the system performance. An adaptive form of user pairing schemes could introduce better SSE in the DL-NOMA system. An adaptive method for user pairing, which enhanced the individual SE was addressed^21^. The suggested work also considered the *i*SIC impact on the data rate. A minimum signal-to-interference plus noise ratio (SINR) was considered to address the fairness and QoS of the user. The study was deficient in a robust theoretical framework. While performing SIC, the other users could intrude on a user’s data, which could be minimized using user pairing schemes. The number of users in an SIC process was minimized through user pairing, thereby reducing the possibility of intruders^22^. A joint optimization of PA and user pairing was considered in the suggested work. The sum secrecy rate was used to analyze the system performance. The study also analyzed the computational complexity of different schemes. The employment of intelligent omni-surface (IOS) with NOMA systems enhanced the SSE and minimized outages. A closed-form expression for the outage probability for the IOS-aided DL-NOMA system under the Rayleigh fading channel was derived^7^. The analysis included user pairing schemes, where the pairing scheme with the maximum separation between users provided the maximum SSE. An optimization method for PA factors was addressed in the suggested work to further enhance the SSE.

### Impact of HI and *i*SIC

The impact of HI in a reconfigurable intelligent surface (RIS)-based DL-NOMA system was discussed^6^. The suggested work discussed the relevance of outage and throughput analysis under HI for the evaluation of the RIS-aided NOMA-based system’s performance. A closed-form expression for the outage probability under HI was derived and compared with simulation results. The results demonstrated that throughput analysis could be utilized to evaluate the system’s real-time SE. While the proposed work was supported by mathematical modeling, the use of fixed PA factors limited the system’s efficiency. The employment of a generalized fading model for the channel analysis was discussed^23^. The suggested work considered the UL-NOMA system under *i*SIC and HI. The impact of *i*SIC and HI on the throughput and SSE of a two-user system was discussed using the results. The concept of the outage floor in UL-NOMA was addressed in the suggested work. It was observed that NOMA had the upper hand over OMA even under *i*SIC and HI. The suggested work analyzed the RIS-assisted DL-NOMA system^24^. A detailed analysis was conducted on the impact of HI. Closed-form expressions for outage probability, SSE, and EE were derived, and the results were validated through simulations. The findings indicated that HI significantly degrades system performance.

A comprehensive outage analysis for the RIS-enabled NOMA system was conducted^25^. A comparative analysis between the NOMA and OMA schemes was performed, with the discussion centered on the implications of various user locations. Additionally, throughput and diversity analyses were carried out to substantiate the robustness of the suggested methodology. Although the theoretical results were aligned with the simulations, the mathematical foundation of the study was found to be lacking in robustness. An optimization approach for the PA factors to minimize the probability of outage under *i*SIC was discussed^26^. The optimization method specifically ensured the QoS of the user located far from the gNB. A combination of NOMA and OMA schemes enhanced the performance in a multi-user DL-NOMA system. The hybrid cooperative NOMA (CNOMA) form, in which user pairs were served through the combination of NOMA and OMA was discussed^27^. The suggested work discussed the employment of IOS and the corresponding outage analysis in the Rayleigh fading environment. The complexity in the multi-user scenario was mitigated through different user pairing strategies, and the performances were compared.

### Channel modeling and fading environments

The capacity analysis for different receiver models under generalized channel models was performed^28^. The results were used to model the Gamma-shadowed Nakagami-*m* fading channel. The role of shaping and shadowing factor of the analytical model over ergodic capacity was illustrated using the results. A systematic analysis of the RIS-aided UL-NOMA system was discussed^29^. The results showed that the user without an LOS connection was more benefited through the usage of RIS connectivity. The suggested work employed the Nakagami-*m* channel model to perform the analysis. Even though the modeling included different scenarios of the system, the results lacked a proper comparison and analysis. The outage analysis of the CNOMA system under the Nakagami-*m* channel was discussed^30^. The suggested work considered DL and UL-NOMA systems and considered different sets of fixed PA factors for the analysis. The outage analysis was conducted for different fading scenarios through the usage of different values of the shape parameter (*m*). The suggested work lacked a PA optimization method and the SSE analysis for understanding performance improvement.

An optimization process for PA factors and the decoding method in the DL-NOMA system under the Nakagami-*m* fading channel was investigated^31^. The closed-form solutions were addressed for outages by considering user fairness. The outage balancing was introduced to provide a balanced fairness condition for every user. The suggested work did not consider UL-NOMA analysis and SSE performance. The generalized approach to evaluate the outage probability for the DL-NOMA system under homogeneous and non-homogeneous channel conditions was discussed^32^. The derived equations could be adjusted to fit the specific channel model by substituting the CDF for each case. Additionally, the study addressed errors arising from channel estimation and *i*SIC operations in NOMA. While the analysis encompassed a range of fading models, it notably lacked a rigorous mathematical foundation to support the generalized model. In the literature survey, we also include works that utilize intermediate nodes or RIS, as they address HI and Nakagami-*m* channel modeling in NOMA systems, both of which are central to our study^24,25,27,29^. These studies underscore the importance of integrating HI effects and generalized channel models into the analysis and performance evaluation of NOMA systems.

### Research gap

Most studies on NOMA focus on either DL or UL configurations. These investigations are performed under the assumption of ideal conditions for SIC and HI, which is practically not feasible. Moreover, these assumptions limit their relevance to real-time 5G and 6G scenarios. Most studies considered basic fading models, such as the Rayleigh or Rician distributions. A generalized fading model analysis can provide significant insights in this area. Furthermore, previous research often examines PA and user pairing as separate issues. A brief overview and comparison of the proposed work and literature are presented in Table 1. The symbols used in this paper and the corresponding descriptions are presented in Table 2.

**Table 1:** Overview and comparison of the proposed method with prior work.

Reference number	DL	UL	*i*SIC	HI	PA opt	Outage	User pairing	SSE	Nakagami-*m*
^4,16,17^	✓				✓	✓		✓	
^6^	✓			✓		✓			
^7^	✓		✓		✓	✓	✓	✓	
^11,30^	✓	✓				✓			✓
^12,25^	✓		✓	✓		✓			
^13^	✓					✓			
^14^	✓	✓			✓		✓		
^15^	✓	✓			✓	✓			
^18^	✓				✓			✓	
^19,20^	✓					✓	✓	✓	
^21^	✓	✓			✓	✓			
^22^	✓				✓		✓	✓	
^23^		✓	✓	✓		✓		✓	✓
^24^	✓			✓		✓		✓	✓
^26^	✓		✓		✓	✓			
^27^	✓		✓	✓	✓	✓	✓	✓	
^28^	✓			✓		✓		✓	✓
^29^		✓				✓			✓
^31^	✓				✓	✓			✓
^32^	✓		✓			✓			✓
This work	✓	✓	✓	✓	✓	✓	✓	✓	✓

**Table 2:** Symbols and descriptions.

Symbols	Description
*m*	Nakagami-*m* shape parameter
*M*	Number of users
$h_{s-j}$	Channel coefficients between gNB and $\text {UD}_j$
$g_{s-j}$	Instantaneous channel po gain between gNB and $\text {UD}_j$
$\delta _{s-j}$	Distance between gNB and $\text {UD}_j$
$\eta$	Path loss parameter
$\alpha _j$	PA factor for $\text {UD}_j$
$P_{t}$	Power budget
$y_{s-j}$, $y_{s}$	Received signals for DL and UL
$\sigma _n^2$	Noise variance
$R_{UD_j}^{DL/UL}$	SE for $\text {UD}_j$ in DL/UL
$S^{DL/UL}_{NOMA}$	SSE in DL/UL
$\tilde{\beta }_j$	Minimum required SE for $\text {UD}_j$
$\Theta _{UE_j}^{DL/UL}$	Channel gain range causing outage for $\text {UD}_j$ in DL/UL
$PO^{DL/UL}_{\text {UD}_j}$	Probability of outage for $\text {UD}_j$ in DL/UL
$\lambda ^2$	Distortion power due to HI
$\lambda$	Standard deviation of HI
$\varepsilon$	*i*SIC distortion parameter
$\Gamma (\cdot )$	Gamma function
$f_X(x)$	PDF
$F_X(x)$	CDF
*E*(.)	Expectation operator
*Var*(.)	Variance operator

## System model

**Fig. 1:**
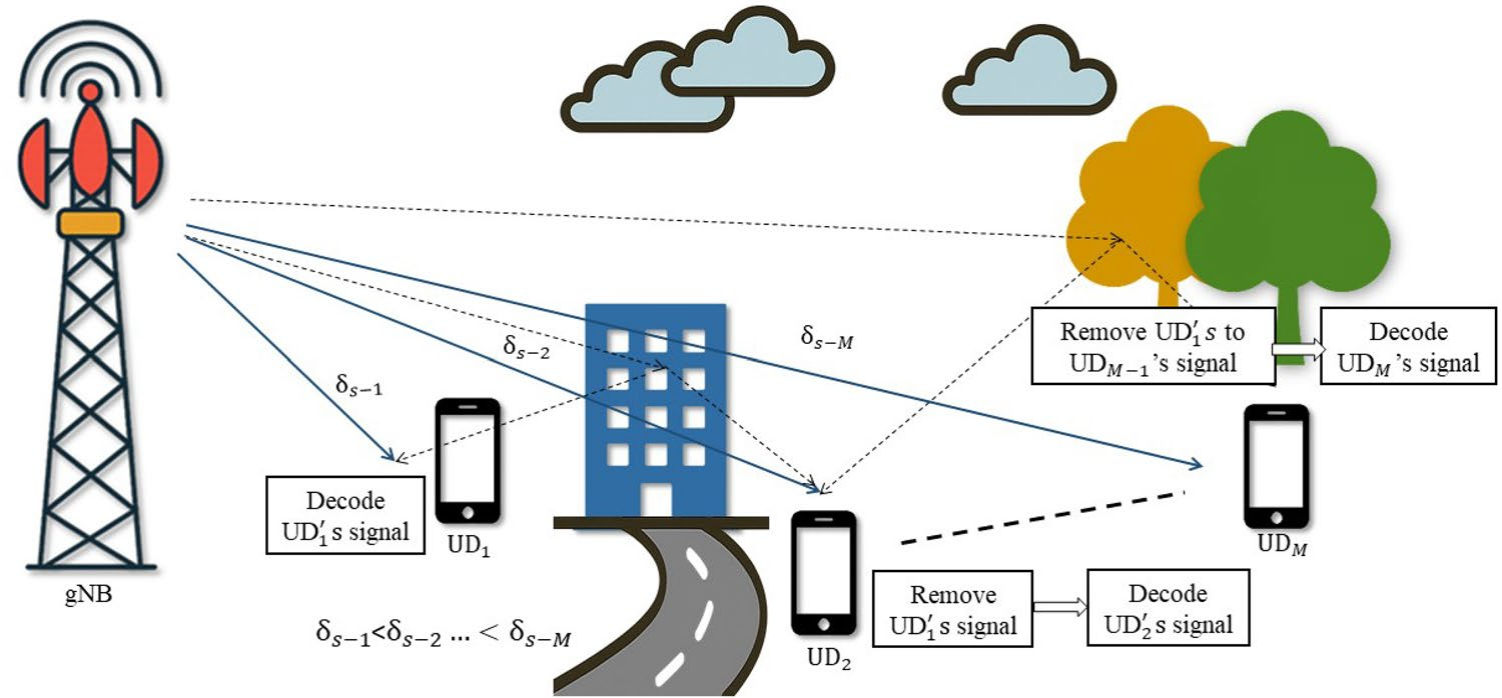
DL-NOMA system model.

**Fig. 2:**
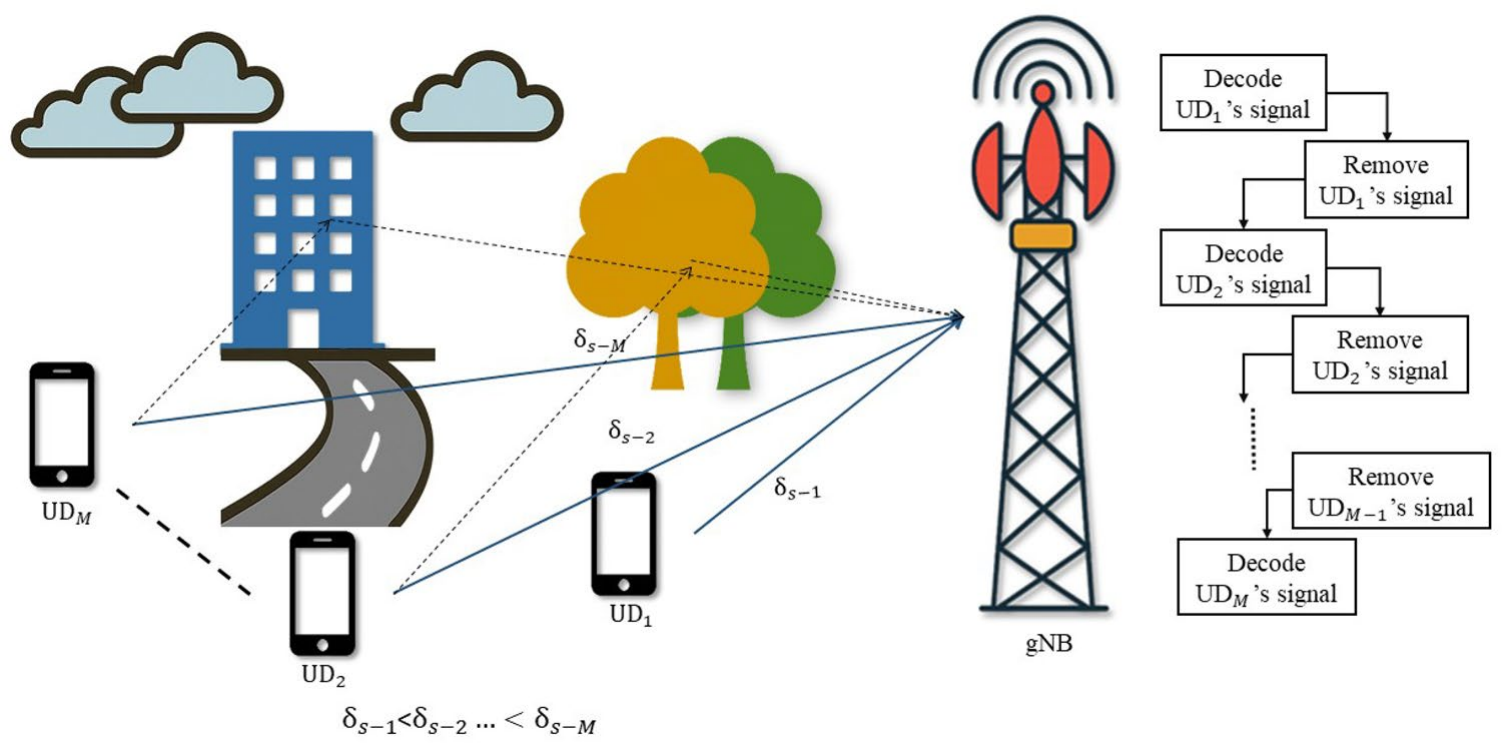
UL-NOMA system model.

Consider a single-cell NOMA system with a gNB and *M* number of user devices. The system models for DL-NOMA and UL-NOMA in a multi-user scenario are illustrated in Figs. 1 and 2, respectively. The user devices are denoted as $\text {UD}_j$, $j=1, 2,...M$. Let the complex Gaussian fading channel coefficients between gNB and $\text {UD}_j$ be denoted by $h_{s-j}$. Consider that the $\text {UD}_j$ is positioned at a distance of $\delta _{s-j}$ from gNB. In the system model, Figs. 1 and 2 are intended to represent a multi-user scenario where $\text {UD}_1$ is the closest to the gNB, followed by $\text {UD}_2$, and so on, up to $\text {UD}_M$ for both UL and DL. This ordering convention is consistently followed throughout the manuscript. The set of channel power gain for $\text {UD}_j$ is denoted as $G_{s-j}$ and the instantaneous channel power gain is denoted as $g_{s-j}$, where $g_{s-j} \in G_{s-j}$. Consider $g_{s-j}= \delta _{s-j}^{-\eta } |h_{s-j}|^2$, where $\eta$ is the path loss exponent. Consider $|h_{s-j}|$ following the Nakagami-*m* distribution, with the shape parameter $m \ge {1/2}$. Hence, the PDF for $G_{s-j}$ is Gamma distributed and it is represented as^11,33^

1 $$\begin{aligned} f_{G_{s-j}} (g_{s-j}) = \frac{m^m}{\Gamma (m)(\delta _{s-j}^{-\eta })^m}g_{s-j}^{m-1}\exp \bigg ({\frac{-mg_{s-j}}{\delta _{s-j}^{-\eta }}}\bigg ) \end{aligned}$$

where $G_{s-j}$ is the random variable and $g_{s-j}$ is the instantaneous realization and $\Gamma (m)$ is the Gamma function. The wireless system model considers DL and UL-NOMA systems with Nakagami-*m* fading channels.

### SSE of DL-NOMA system

Consider the DL-NOMA system for $M=2$, where $\text {UD}_1$ is located close to the gNB at a distance of $\delta _{s-1}$ and $\text {UD}_2$ is away from it at a distance of $\delta _{s-2}$, that is, $\delta _{s-1}<\delta _{s-2}$ and $E\{G_{s-1}\}> E\{G_{s-2}\}$. The user devices are differentiated according to their average channel power gain. Hence, for fairness the percentage of power allocated to $\text {UD}_2$ is higher than $\text {UD}_1$, that is, $\alpha _1<\alpha _2$. Here, $\alpha _j$ is the PA factor for $\text {UD}_j$, which follows condition $\sum _{j=1}^2\alpha _j=1$. Let the transmission and reception power budget be denoted as $P_t$. Consider $x_{s-j}$ as the information signal corresponding to $\text {UD}_j$. The composite transmitted signal for DL-NOMA is represented as^34^,

2 $$\begin{aligned} x_{DL}= \sum _{j=1}^{2}\sqrt{\alpha _j P_t}x_{s-j} \end{aligned}$$

Let $y_{s-j}$ be the corresponding received signal at $\text {UD}_j$ and it is represented as,

3 $$\begin{aligned} y_{s-j}= \sqrt{\delta _{s-j}^{-\eta } |h_{s-j}|^2} x_{DL}+ \omega _{j} \end{aligned}$$

where $\omega _{j}\sim \mathcal {C}\mathcal {N}(0,\sigma _n^2)$ denotes the additive white Gaussian noise (AWGN) which affects $x_{DL}$, and $\sigma _n^2$ is the noise power. Substituting (2) in (3) results,

4 $$\begin{aligned} y_{s-j} = \sqrt{\delta _{s-j}^{-\eta } |h_{s-j}|^2} \sum _{j=1}^{2}\sqrt{\alpha _j P_t}x_{s-j}+ \omega _{j} \end{aligned}$$

As $\text {UD}_1$ is positioned close to the gNB, it performs SIC to decode its information. During the process, $\text {UD}_1$ decodes and removes the signal corresponding to $\text {UD}_2$ within $y_{s-j}$ by treating the remaining signal as interference. The SE achievable at $\text {UD}_1$ for $\text {UD}_2$’s signal is expressed using Shannon’s capacity equation as,

5 $$\begin{aligned} R_{UD_1}^{UD_2}=\log _2\bigg (1+\frac{g_{s-1} \alpha _2 \rho _n}{g_{s-1} \alpha _1 \rho _n +1} \bigg ) \end{aligned}$$

where $\frac{g_{s-1} \alpha _2 \rho _n}{g_{s-1} \alpha _1 \rho _n +1}$ is the the SINR at $\text {UD}_1$, for $\text {UD}_2$’s signal and $\rho _n=P_t/\sigma _n^2$. $R_{UD_1}^{UD_2}$ is used to derive the probability of outage for $\text {UD}_1$. After removing $\text {UD}_2$’s signal, $\text {UD}_1$ decodes its information from the remaining signal. A high percentage of power is allocated to $\text {UD}_2$; hence, $\text {UD}_2$ decodes its information directly by considering the information of $\text {UD}_1$ as interference. Thus, the general form of SE achievable at $\text {UD}_j$ to decode $x_j$ is expressed as,

6 $$\begin{aligned} R_{s-j}^{DL}={\left\{ \begin{array}{ll} \log _2(1+g_{s-1} \alpha _1 \rho _n), & \text {for }j=1.\\ \log _2\bigg (1+\frac{g_{s-2} \alpha _2 \rho _n}{g_{s-2}\alpha _1 \rho _n +1}\bigg ), & \text {for }j=2. \end{array}\right. } \end{aligned}$$

#### Remark

This equation captures the fundamental power-domain multiplexing of NOMA. $\text {UD}_1$, being the strong user, decodes its own data after SIC, hence its SE depends only on its allocated power $\alpha _1$ and channel gain. $\text {UD}_2$, the weak user, decodes directly but suffers from interference caused by $\text {UD}_1$’s signal, which appears in the denominator of its SINR expression.

The achievable SSE in the DL-NOMA system is the sum of individual SE, and it is expressed as,

7 $$\begin{aligned} S_{NOMA}^{DL} = \sum _{j=1}^2 R_{s-j}^{DL} \end{aligned}$$

### SSE of DL-NOMA with HI and *i*SIC

The HI is a substantial challenge in wireless systems. Understanding and simulating HI is crucial for designing future wireless networks. The HI in the NOMA system arises due to the imperfections in the practical transceivers, which degrade the system’s performance. Non-idealities in transceiver components such as power amplifier nonlinearities, phase noise, I/Q imbalance, and quantization errors, which introduce distortion into the transmitted or received signals results in HI. The received signal at $\text {UD}_j$ in DL-NOMA is rewritten by including HI is expressed as^6^,

8 $$\begin{aligned} y_{s-j} = \sqrt{\delta _{s-j}^{-\eta } |h_{s-j}|^2} \left( \sum _{j=1}^{2}\sqrt{\alpha _j P_t}x_{s-j}+\Omega _s\right) + \Omega _{r}+\omega _{j} \end{aligned}$$

where $\Omega _{s}$ and $\Omega _{r}$ are the HI from the transmitter and the receiver, which is modeled as: $\Omega _{s}\sim \mathcal {C}\mathcal {N}(0,\lambda _s^2P_t)$ and $\Omega _{r}\sim \mathcal {C}\mathcal {N}(0,|h_{s-j}|^2\lambda _r^2P_t)$^35^. Unlike AWGN, which is independent of the transmitted signal power and typically modelled as constant-variance thermal noise with variance $\sigma _n^2$, the distortion caused by HI is signal-dependent. Its power increases with the transmit signal power. This impairment noise is often modelled, for analytical tractability, as zero-mean complex Gaussian with variance proportional to the transmit power, i.e., $\lambda _s^2$ at the transmitter and $\lambda _r^2$ at the receiver, where $\lambda _s$ and $\lambda _r$ are the terms that represent the severity of the impairments. According to the central limit theorem (CLT), the sum of these large number of small, independent distortion sources tends toward a Gaussian distribution, making the Gaussian assumption a reasonable approximation. Furthermore, since wireless baseband signals are inherently complex, comprising in-phase (I) and quadrature (Q) components, the impairments affect both components. Thus, modelling the HI as complex Gaussian noise ensures mathematical consistency with baseband signal processing. Hence, the combined HI noise is defined as, $\Omega \triangleq \Omega _s+\Omega _r/h_{s-j}$, and it follows a complex Gaussian distribution $\Omega \sim \mathcal {C}\mathcal {N}(0,\lambda ^2P_t)$, where $\lambda ^2=\lambda _s^2+\lambda _r^2$. This follows from the closure property of Gaussian distributions under linear combinations. Specifically, the variance of the sum is the sum of the individual variances. Thus, the aggregate impact of HI is modelled as a Gaussian distribution, providing a robust framework for characterizing these effects^6,35,36^. The SE achievable at $\text {UD}_1$ for $\text {UD}_2$’s signal including HI noise is expressed as,

9 $$\begin{aligned} R_{UD_1}^{im-UD_2}=\log _2\bigg (1+\frac{g_{s-1} \alpha _2 \rho _n}{g_{s-1} \alpha _1 \rho _n +g_{s-1}\lambda ^2\rho _n+1} \bigg ) \end{aligned}$$

Here ‘$im$’ in the terms indicates the imperfections in the system. The denominator in (9) contains an additional term $g_{s-1}\lambda ^2\rho _n$ representing the effective noise power increase due to HI. This shows that HI degrades performance even before accounting for *i*SIC. In an ideal scenario, the SIC process completely removes the residual information from $\text {UD}_2$. However, in practical situations, *i*SIC is unavoidable and introduces outages during signal detection. The *i*SIC affects the user device that performs SIC; here, it is $\text {UD}_1$. The general form of SE achievable at $\text {UD}_j$ to decode $x_j$ including HI and *i*SIC is expressed as,

10 $$\begin{aligned} R_{s-j}^{im-DL}={\left\{ \begin{array}{ll} \log _2\bigg (1+\frac{g_{s-1} \alpha _1 \rho _n}{\varepsilon g_{s-1}\alpha _2\rho _n+g_{s-1}\lambda ^2\rho _n+1}\bigg ), & \text {for }j=1.\\ \log _2\bigg (1+\frac{g_{s-2} \alpha _2 \rho _n}{g_{s-2}\alpha _1 \rho _n +g_{s-2}\lambda ^2\rho _n +1}\bigg ), & \text {for }j=2. \end{array}\right. } \end{aligned}$$

#### Remark

Equation (10) shows how the achievable SE for each user in DL-NOMA is degraded by hardware distortion power $(\lambda ^2)$ and residual interference from *i*SIC $(\varepsilon )$ . A larger $\varepsilon$ increases the interference term in the denominator for $\text {UD}_1$, significantly reducing its SE. In contrast, $\text {UD}_2$ is unaffected by $\varepsilon$ because it does not perform SIC.

The *i*SIC distortion parameter $(\varepsilon )$ in (10) represents the fraction of residual interference remaining after SIC. Its can ideally take the range $0\le \varepsilon \le 1$. However, in alignment with common practice in the literature, we consider $\varepsilon =0$ and $\varepsilon =0.1$ for the analysis, corresponding to 0% and 10% of the interfering signal power^26^. This reflects realistic distortion levels encountered in practical NOMA systems. A value of $\varepsilon =0$ indicates perfect SIC, while higher values model increasing levels of imperfection.

The achievable SSE in the DL-NOMA system is the sum of individual SE, and it is expressed as,

11 $$\begin{aligned} S_{NOMA}^{im-DL} = \sum _{j=1}^2 R_{s-j}^{im-DL} \end{aligned}$$

### SSE of UL-NOMA system

Consider the UL-NOMA system for $M=2$, where $\text {UD}_1$ is located close to the gNB at a distance of $\delta _{s-1}$ and $\text {UD}_2$ is away from it at a distance of $\delta _{s-2}$. The PA factor considered for $\text {UD}_1$ is higher than $\text {UD}_2$, that is, $\alpha _1>\alpha _2$. Let $P_t$ represent the UL-NOMA power budget and $x_j$ represent the data transmitted from all $\text {UD}_j$. In UL-NOMA, gNB receives a superposed signal from all $\text {UD}_j$ and is expressed as^37^,

12 $$\begin{aligned} y_{s}= \Sigma _{j=1}^{2}\sqrt{\delta _{s-j}^{-\eta } |h_{s-j}|^2}\sqrt{\alpha _j P_t} x_j + \omega _{s} \end{aligned}$$

Since $y_{s}$ consists of data from $\text {UD}_1$ with higher signal power than $\text {UD}_2$, gNB decodes $\text {UD}_1$’s data directly by considering the rest of the signal as interference and noise. After detecting $\text {UD}_1$’s data, gNB removes it from $y_{s}$, and $\text {UD}_2$’s data will be detected from the remaining signal. Thus, the general form of SE achievable at $\text {UD}_j$ to decode $x_j$ at gNB is expressed as,

13 $$\begin{aligned} R_{s-j}^{UL}={\left\{ \begin{array}{ll} \log _2\bigg (1+\frac{g_{s-1} \alpha _1 \rho _n}{g_{s-2} \alpha _2 \rho _n+1}\bigg ), & \text {for }j=1.\\ \log _2(1+g_{s-2} \alpha _2 \rho _n), & \text {for }j=2. \end{array}\right. } \end{aligned}$$

The achievable SSE in the UL-NOMA system is expressed as,

14 $$\begin{aligned} S_{NOMA}^{UL} = \sum _{j=1}^2 R_{s-j}^{UL} \end{aligned}$$

### SSE of UL-NOMA with HI and *i*SIC

The composite signal, including HI received at gNB, is expressed as,

15 $$\begin{aligned} y_{s}= \Sigma _{j=1}^{2}\sqrt{\delta _{s-j}^{-\eta } |h_{s-j}|^2}\left( \sqrt{\alpha _j P_t} x_j +\Omega _{j}\right) +\Omega _s+ \omega _{s} \end{aligned}$$

where $\Omega _{j}$ is the HI from $\text {UD}_j$. The aggregate HI is defined in the same way as DL-NOMA, and the general form of SE achievable at $\text {UD}_j$ to decode $x_j$ at gNB, including HI and *i*SIC is expressed as,

16 $$\begin{aligned} R_{s-j}^{im-UL}={\left\{ \begin{array}{ll} \log _2\bigg (1+\frac{g_{s-1} \alpha _1 \rho _n}{g_{s-2} \alpha _2 \rho _n + g_{s-1} \alpha _1\lambda ^2\rho _n + g_{s-2} \alpha _2\lambda ^2\rho _n+1}\bigg ), & \text {for }j=1.\\ \log _2\left( 1+\frac{g_{s-2} \alpha _2 \rho _n}{\varepsilon g_{s-1}\alpha _1\rho _n + \varepsilon g_{s-1}\alpha _1\rho _n \lambda ^2+ g_{s-2}\alpha _2\lambda ^2\rho _n+1}\right) , & \text {for }j=2. \end{array}\right. } \end{aligned}$$

#### Remark

In UL-NOMA, $\varepsilon$ appears in $\text {UD}_2$’s rate expression because the gNB need to remove $\text {UD}_1$’s strong signal before decoding $\text {UD}_2$’s data. Any residual interference directly limits $\text {UD}_2$’s achievable SE, while $\text {UD}_1$’s SE is unaffected by $\varepsilon$ but still degrades with HI.

The achievable SSE in the UL-NOMA system is expressed as,

17 $$\begin{aligned} S_{NOMA}^{im-UL} = \sum _{j=1}^2 R_{s-j}^{im-UL} \end{aligned}$$

## Outage analysis

The probability of outage for DL-NOMA and UL-NOMA under Nakagami-*m* fading channel conditions is derived in this section. The initial subsection considers the perfect SIC (*p*SIC) process and negligible HI for the derivation. The following subsection explores scenarios involving *i*SIC and HI.

### Outage probability analysis for DL-NOMA

The outage condition for $\text {UD}_j$ occurs when SE drops below a specified minimum threshold. Let ${\tilde{\beta }_j}$ be the minimum SE required for $\text {UD}_j$. In order for $\text {UD}_1$ to carry out SIC, $R_{UD_1}^{UD_2}$ must be greater than ${\tilde{\beta }_2}$. Hence, for $\text {UD}_1$, the outage scenario is defined by the combination of two conditions, denoted as $(R_{s-1}^{DL}<{\tilde{\beta }_1})\cup (R_{UD_1}^{UD_2}<\tilde{\beta }_{2})$. By substituting (5) and (6) for $j=1$, into the given conditions, the two distinct ranges for $g_{s-1}$ are formulated. The outage condition at $\text {UD}_1$ is defined in terms of $g_{s-1}$ by selecting the maximum value of these ranges and is expressed as,

18 $$\begin{aligned} g_{s-1}<\underbrace{\max { \left\{ \frac{\beta _1}{\alpha _1 \rho _n}, \frac{\beta _{2}}{(\alpha _2-\alpha _1{\beta _{2}}) \rho _n} \right\} }}_{\Theta _{s-1}^{DL}} \end{aligned}$$

where $\beta _1= 2^{\tilde{\beta }_1}-1$ and $\beta _{2} = 2^{\tilde{\beta }_{2}}-1$. In the case of $\text {UD}_2$, the outage condition is defined as $(R_{s-2}^{DL}<{\tilde{\beta }_2})$. The outage condition for $\text {UD}_2$ in terms of $g_{s-2}$ is derived by substituting (6) for $j=2$ into the given criteria, and it is represented as,

19 $$\begin{aligned} g_{s-2}<\underbrace{\frac{\beta _2}{(\alpha _2-\alpha _1 \beta _2)\rho _n}}_{\Theta _{s-2}^{DL}} \end{aligned}$$

The outage probability for $\text {UD}_j$ is determined by integrating the PDF of $G_{s-j}$ over the derived limit, $\Theta _{s-j}^{DL}$, which is expressed as,

20 $$\begin{aligned} PO_{UD_j}^{DL}= \int _{0}^{\Theta _{s-j}^{DL}}f_{G_{s-j}} (g_{s-j})dg_{s-j},\;\;\;j=1,2 \end{aligned}$$

#### Remark

The outage probability is obtained by integrating the channel gain PDF up to a threshold $\Theta _{s-j}^{DL}$, which depends on both PA and SE requirements. A higher SE threshold or lower PA increases $\Theta _{s-j}^{DL}$, leading to a higher outage probability.

By substituting (1) in (20) results,

21 $$\begin{aligned} PO_{UD_j}^{DL}= \int _{0}^{\Theta _{s-j}^{DL}}\frac{m^m}{\Gamma (m)(\delta _{s-j}^{-\eta })^m}g_{s-j}^{m-1}\exp \bigg ({\frac{-mg_{s-j}}{\delta _{s-j}^{-\eta }}}\bigg )dg_{s-j},\;\;\;j=1,2 \end{aligned}$$

Let $u={\frac{mg_{s-j}}{\delta _{s-j}^{-\eta }}}$, then $dg_{s-j}=\frac{\delta _{s-j}^{-\eta }}{m}du$. The lower limit for integration is zero, and the upper limit changes to ${\frac{m\Theta _{s-j}^{DL}}{\delta _{s-j}^{-\eta }}}$. Substituting and rearranging (21) results,

22 $$\begin{aligned} PO_{UD_j}^{DL}= \frac{1}{\Gamma (m)}\int _{0}^{{\frac{m\Theta _{s-j}^{DL}}{\delta _{s-j}^{-\eta }}}}u^{m-1}\exp (-u)du,\;\;\;j=1,2 \end{aligned}$$

The probability of outage of $\text {UD}_j$ in the DL-NOMA system is obtained by comparing (22) with the incomplete Gamma function, which is defined as $\gamma (s,t)= \int _{0}^{t}u^{s-1}\exp (-u)du$ and it results,

23 $$\begin{aligned} PO_{UD_j}^{DL}= \frac{\gamma \bigg (m,{\frac{m\Theta _{s-j}^{DL}}{\delta _{s-j}^{-\eta }}}\bigg )}{\Gamma (m)},\;\;\;j=1,2 \end{aligned}$$

Here (23) represents the closed-form expression for the probability of outage for the DL-NOMA system with $M=2$. The expression includes the lower incomplete Gamma function defined in terms of $m, \Theta _{s-j}^{DL}$, and the average channel gain and the Gamma distribution (GD) function defined in terms of *m*.

#### Remark

Equation (23) expresses the outage probability in terms of the lower incomplete Gamma function, directly relating the result to the Nakagami-*m* fading severity parameter. A higher *m* (less severe fading) leads to smaller values of the incomplete Gamma term, thereby reducing outage probability.

### Outage probability analysis in DL-NOMA with HI and *i*SIC

For $\text {UD}_1$, the outage scenario under HI and *i*SIC is defined by the conditions, denoted as $(R_{s-1}^{im-DL}<{\tilde{\beta }_1})\cup (R_{UD_1}^{im-UD_2}<\tilde{\beta }_{2})$. By substituting (9) and (10) for $j=1$, into the given conditions, the two distinct ranges for $g_{s-1}$ are formulated. The outage condition at $\text {UD}_1$ is defined in terms of $g_{s-1}$ by selecting the maximum value of these ranges and is expressed as,

24 $$\begin{aligned} g_{s-1}<\underbrace{\max { \left\{ \frac{\beta _1}{(\alpha _1-\varepsilon \alpha _2 \beta _1-\lambda ^2\beta _1)\rho _n}, \frac{\beta _{2}}{(\alpha _2-\alpha _1{\beta _{2}}-\lambda ^2\beta _2) \rho _n} \right\} }}_{\Theta _{s-1}^{im-DL}} \end{aligned}$$

In the case of $\text {UD}_2$, the outage condition is defined as $(R_{s-2}^{im-DL}<{\tilde{\beta }_2})$. The outage condition for $\text {UD}_2$ in terms of $g_{s-2}$ is derived by substituting (10) for $j=2$ into the given criteria, and it is represented as follows.

25 $$\begin{aligned} g_{s-2}<\underbrace{\frac{\beta _2}{(\alpha _2-\alpha _1 \beta _2-\lambda ^2\beta _2)\rho _n}}_{\Theta _{s-2}^{im-DL}} \end{aligned}$$

The outage probability for $\text {UD}_j$ is determined by integrating the PDF of $G_{s-j}$ over the derived limit, $\Theta _{s-j}^{im-DL}$, which is expressed as,

26 $$\begin{aligned} PO_{UD_j}^{im-DL}= \int _{0}^{\Theta _{s-j}^{im-DL}}f_{G_{s-j}} (g_{s-j})dg_{s-j},\;\;\;j=1,2 \end{aligned}$$

Here, (26) is similar to (20), with changes in the limits of integration. Hence, the final expression for $\text {UD}_j$ under HI and *i*SIC is obtained by replacing $\Theta _{s-j}^{DL}$ in (23) with $\Theta _{s-j}^{im-DL}$, and is expressed as,

27 $$\begin{aligned} PO_{UD_j}^{im-DL}= \frac{\gamma \bigg (m,{\frac{m\Theta _{s-j}^{im-DL}}{\delta _{s-j}^{-\eta }}}\bigg )}{\Gamma (m)},\;\;\;j=1,2 \end{aligned}$$

Compared with (23), (27) incorporates the derived limit $\Theta _{s-j}^{im-DL}$ that account for HI and *i*SIC. Both impairments effectively increasing the probability of outage.

### Outage probability analysis in UL-NOMA

In UL-NOMA, the data outage for $\text {UD}_1$ at gNB takes place when the corresponding SE drops below a specified minimum threshold. Hence, the condition for outage for $\text {UD}_1$ at gNB is denoted as $R_{s-1}^{UL}<\tilde{\beta }_1$, where $\tilde{\beta }_1$ is the minimum required SE for $\text {UD}_1$. The condition for the probability of outage in terms of $g_{s-1}$ is determined by substituting (13) for $j=1$ into the condition, and it results,

28 $$\begin{aligned} g_{s-1}<\underbrace{\frac{\beta _1}{\alpha _1\rho _n}+\frac{\beta _1\alpha _2}{\alpha _1}g_{s-2}}_{\Theta _{s-1}^{UL}} \end{aligned}$$

where ${\Theta _{s-1}^{UL}}$ is defined in terms of $g_{s-2}$, which varies from zero to infinity. The outage threshold for $\text {UD}_1$ in UL depends linearly on $g_{s-2}$, the gain of the weak user. This coupling reflects the interference from the other user’s transmission, a characteristic feature of NOMA. The gNB performs SIC to decode $\text {UD}_2$’s data, hence the condition for outage for $\text {UD}_2$ consists of two conditions, denoted as $R_{s-1}^{UL}<\tilde{\beta }_1$ and $R_{s-2}^{UL}<\tilde{\beta }_2$, where $\tilde{\beta }_2$ is the minimum required SE for $\text {UD}_2$. When either of these conditions is satisfied, $\text {UD}_2$ will be in outage. The corresponding ranges for $g_{s-1}$ and $g_{s-2}$ is determined by substituting (13) into the condition, and it results,

29 $$\begin{aligned} g_{s-1}<\underbrace{\frac{\beta _1}{\alpha _1\rho _n}+\frac{\beta _1\alpha _2}{\alpha _1}g_{s-2}}_{\Theta _{s-1}^{UL}},\; g_{s-2}<\underbrace{\frac{\beta _2}{\alpha _2\rho _n}}_{\Theta _{s-2}^{UL}} \end{aligned}$$

Based on the conditions outlined in (28), the outage probability of $\text {UD}_1$ is defined and articulated as,

30 $$\begin{aligned} PO_{UD_1}^{UL}=\int _{0}^{\infty }{\underbrace{\left\{ {\int ^{{\Theta _{s-1}^{UL}}}_{0} f_{G_{s-1}}(g_{s-1})dg_{s-1}}\right\} }_{F^{UL-1}_{G_{s-1}} (g_{s-1})} f_{G_{s-2}} (g_{s-2})}dg_{s-2} \end{aligned}$$

The expression for ${F^{UL-1}_{G_{s-1}} (g_{s-1})}$ is similar to (20) with changes in the integration limit. Hence, the result is obtained by replacing $\Theta _{s-j}^{DL}$ with $\Theta _{s-1}^{UL}$ in (23), and it results,

31 $$\begin{aligned} {F^{UL-1}_{G_{s-1}} (g_{s-1})} = \frac{\gamma \bigg (m,{\frac{m\Theta _{s-1}^{UL}}{\delta _{s-1}^{-\eta }}}\bigg )}{\Gamma (m)} \end{aligned}$$

For further derivation, the lower incomplete Gamma function is represented in terms of exponential series, which is generally represented as^38^,

32 $$\begin{aligned} \gamma (s,t) = (s-1)!\left( 1-\exp (-t)\left( 1+t+\frac{t^2}{2!}+\frac{t^3}{3!}+...\frac{t^{s-1}}{(s-1)!}\right) \right) \end{aligned}$$

where *s* is an integer. The lower incomplete Gamma function in (31) is expanded in terms of exponential series as in (32), and it is expressed as,

33 $$\begin{aligned} {F^{UL-1}_{G_{s-1}} (g_{s-1})}= & \frac{(m-1)!}{\Gamma (m)}\bigg (1-\exp {\left( -{\frac{m\Theta _{s-1}^{UL}}{\delta _{s-1}^{-\eta }}}\right) }\bigg (1+{\left( {\frac{m\Theta _{s-1}^{UL}}{\delta _{s-1}^{-\eta }}}\right) }+\frac{1}{2!}{\left( {\frac{m\Theta _{s-1}^{UL}}{\delta _{s-1}^{-\eta }}}\right) }^2\nonumber \\ & +\frac{1}{3!}{\left( {\frac{m\Theta _{s-1}^{UL}}{\delta _{s-1}^{-\eta }}}\right) }^3+...\frac{1}{(m-1)!}{\left( {\frac{m\Theta _{s-1}^{UL}}{\delta _{s-1}^{-\eta }}}\right) }^{m-1}\bigg )\bigg ) \end{aligned}$$

where *m* is considered as an integer and $\Gamma (m)=(m-1)!$. Replace $\Theta _{s-1}^{UL}$ using (28) results,

34 $$\begin{aligned} \begin{aligned} {F^{UL-1}_{G_{s-1}} (g_{s-1})}&= 1-\exp {\left( -{\frac{m}{\delta _{s-1}^{-\eta }}} \left( {\frac{\beta _1}{\alpha _1\rho _n}+\frac{\beta _1\alpha _2}{\alpha _1}g_{s-2}}\right) \right) } \bigg (1+{\left( {\frac{m}{\delta _{s-1}^{-\eta }}}\right) \left( {\frac{\beta _1}{\alpha _1\rho _n}+\frac{\beta _1\alpha _2}{\alpha _1}g_{s-2}}\right) }\\&+\frac{1}{2!}{\left( {\frac{m}{\delta _{s-1}^{-\eta }}}\right) }^2\left( {\frac{\beta _1}{\alpha _1\rho _n}+\frac{\beta _1\alpha _2}{\alpha _1}g_{s-2}}\right) ^2 \\&+\frac{1}{3!}{\left( {\frac{m}{\delta _{s-1}^{-\eta }}}\right) }^3\left( {\frac{\beta _1}{\alpha _1\rho _n}+\frac{\beta _1\alpha _2}{\alpha _1}g_{s-2}}\right) ^3\\&+...\frac{1}{(m-1)!}{\left( {\frac{m}{\delta _{s-1}^{-\eta }}}\right) }^{m-1}\left( {\frac{\beta _1}{\alpha _1\rho _n}+\frac{\beta _1\alpha _2}{\alpha _1}g_{s-2}}\right) ^{m-1}\bigg ) \end{aligned} \end{aligned}$$

Applying binomial expansion and arranging different powers of $g_{s-1}$ together, results,

35 $$\begin{aligned} \begin{aligned} {F^{UL-1}_{G_{s-1}} (g_{s-1})}&= 1-\exp {\left( -{\frac{m}{\delta _{s-1}^{-\eta }}} \left( {\frac{\beta _1}{\alpha _1\rho _n} +\frac{\beta _1\alpha _2}{\alpha _1}g_{s-2}}\right) \right) }\\&\bigg (\sum _{n=0}^{m-1}\frac{1}{n!}\left( {\frac{m}{\delta _{s-1}^{-\eta }}}\right) ^n \bigg (\frac{\beta _1}{\alpha _1\rho _n}\bigg )^n\frac{n!}{0!(n-0)!} \\ &+\left( \frac{\beta _1\alpha _2}{\alpha _1}\right) g_{s-2}\sum _{n=1}^{m-1} \left( \frac{1}{n!}\right) \left( {\frac{m}{\delta _{s-1}^{-\eta }}}\right) ^n\bigg (\frac{\beta _1}{\alpha _1\rho _n}\bigg )^{n-1}\frac{n!}{1!(n-1)!} \\ &+\left( \frac{\beta _1\alpha _2}{\alpha _1}\right) ^2g_{s-2}^2\sum _{n=2}^{m-1}\left( \frac{1}{n!}\right) \left( {\frac{m}{\delta _{s-1}^{-\eta }}}\right) ^n\bigg (\frac{\beta _1}{\alpha _1\rho _n}\bigg )^{n-2}\frac{n!}{2!(n-2)!} \\ &+\left( \frac{\beta _1\alpha _2}{\alpha _1}\right) ^3g_{s-2}^3\sum _{n=3}^{m-1}\left( \frac{1}{n!}\right) \left( {\frac{m}{\delta _{s-1}^{-\eta }}}\right) ^n\bigg (\frac{\beta _1}{\alpha _1\rho _n}\bigg )^{n-3}\frac{n!}{3!(n-3)!} \\ &...\; +\left( \frac{\beta _1\alpha _2}{\alpha _1}\right) ^{m-1}g_{s-2}^{m-1}\sum _{n=m-1}^{m-1}\left( \frac{1}{n!}\right) \left( {\frac{m}{\delta _{s-1}^{-\eta }}}\right) ^n\\&\bigg (\frac{\beta _1}{\alpha _1\rho _n}\bigg )^{n-(m-1)}\frac{n!}{(m-1)!(n-(m-1))!} \bigg ) \end{aligned} \end{aligned}$$

Substituting (1) for $j=2$, and (35) in (30), then expanding the terms and applying the integral results,

36 $$\begin{aligned} \begin{aligned} PO_{UD_1}^{UL}&=\frac{m^m}{\Gamma (m)(\delta _{s-2}^{-\eta })^m}\int _{0}^{\infty } g_{s-2}^{m-1}\exp \bigg ({\frac{-mg_{s-2}}{\delta _{s-2}^{-\eta }}}\bigg )dg_{s-2} \\ &-\frac{m^m}{\Gamma (m)(\delta _{s-2}^{-\eta })^m}\exp {\bigg ({\frac{-m\beta _1}{\delta _{s-1}^{-\eta }\alpha _1\rho _n}}\bigg )}\bigg (\sum _{n=0}^{m-1}\bigg (\frac{m}{{\delta _{s-1}^{-\eta }}}\bigg )^n\bigg (\frac{\beta _1}{\alpha _1\rho _n}\bigg )^n\frac{1}{0!(n-0)!}\bigg ) \\ &\int _{0}^{\infty } g_{s-2}^{m-1}\exp \left( {\left( \frac{-m}{\delta _{s-2}^{-\eta }}-{\frac{m\beta _1\alpha _2}{\delta _{s-1}^{-\eta }\alpha _1}}\right) g_{s-2}}\right) dg_{s-2} \\ &-\frac{m^m}{\Gamma (m)(\delta _{s-2}^{-\eta })^m}\left( \frac{\beta _1\alpha _2}{\alpha _1}\right) \exp {\bigg ({\frac{-m\beta _1}{\delta _{s-1}^{-\eta }\alpha _1\rho _n}}\bigg )}\bigg (\sum _{n=1}^{m-1}\bigg (\frac{m}{\delta _{s-1}^{-\eta }}\bigg )^n\bigg (\frac{\beta _1}{\alpha _1\rho _n}\bigg )^{n-1}\frac{1}{1!(n-1)!}\bigg )\\ &\int _{0}^{\infty }g_{s-2}^{m}\exp \left( {\left( \frac{-m}{\delta _{s-2}^{-\eta }}-{\frac{m\beta _1\alpha _2}{\delta _{s-1}^{-\eta }\alpha _1}}\right) g_{s-2}}\right) dg_{s-2} \\ &-\frac{m^m}{\Gamma (m)(\delta _{s-2}^{-\eta })^m}\left( \frac{\beta _1\alpha _2}{\alpha _1}\right) ^2\exp {\bigg ({\frac{-m\beta _1}{\delta _{s-1}^{-\eta }\alpha _1\rho _n}}\bigg )}\bigg (\sum _{n=2}^{m-1}\bigg (\frac{m}{\delta _{s-1}^{-\eta }}\bigg )^n\bigg (\frac{\beta _1}{\alpha _1\rho _n}\bigg )^{n-2}\frac{1}{2!(n-2)!}\bigg )\\ &\int _{0}^{\infty }g_{s-2}^{m+1}\exp \left( {\left( \frac{-m}{\delta _{s-2}^{-\eta }}-{\frac{m\beta _1\alpha _2}{\delta _{s-1}^{-\eta }\alpha _1}}\right) g_{s-2}}\right) dg_{s-2} \\ &-\frac{m^m}{\Gamma (m)(\delta _{s-2}^{-\eta })^m}\left( \frac{\beta _1\alpha _2}{\alpha _1}\right) ^3\exp {\bigg ({\frac{-m\beta _1}{\delta _{s-1}^{-\eta }\alpha _1\rho _n}}\bigg )}\bigg (\sum _{n=3}^{m-1}\bigg (\frac{m}{\delta _{s-1}^{-\eta }}\bigg )^n\bigg (\frac{\beta _1}{\alpha _1\rho _n}\bigg )^{n-3}\frac{1}{3!(n-3)!}\bigg )\\ &\int _{0}^{\infty }g_{s-2}^{m+2}\exp \left( {\left( \frac{-m}{\delta _{s-2}^{-\eta }}-{\frac{m\beta _1\alpha _2}{\delta _{s-1}^{-\eta }\alpha _1}}\right) g_{s-2}}\right) dg_{s-2} \\ &...\; -\frac{m^m}{\Gamma (m)(\delta _{s-2}^{-\eta })^m}\left( \frac{\beta _1\alpha _2}{\alpha _1}\right) ^{m-1}\exp {\bigg ({\frac{-m\beta _1}{\delta _{s-1}^{-\eta }\alpha _1\rho _n}}\bigg )}\sum _{n=m-1}^{m-1}\frac{m^n}{n!(\delta _{s-1}^{-\eta })^n}\\ &\int _{0}^{\infty }g_{s-2}^{2(m-1)}\exp \left( {\left( \frac{-m}{\delta _{s-2}^{-\eta }}-{\frac{m\beta _1\alpha _2}{\delta _{s-1}^{-\eta }\alpha _1}}\right) g_{s-2}}\right) dg_{s-2} \end{aligned} \end{aligned}$$

By performing the calculations and incorporating the integration of (36), the resulting equation is expressed as,

37 $$\begin{aligned} \begin{aligned} PO_{UD_1}^{UL}&=\frac{m^m}{\Gamma (m)(\delta _{s-2}^{-\eta })^m}\times \frac{\Gamma (m)(\delta _{s-2}^{-\eta })^m}{m^m} \\ &-\frac{m^m}{\Gamma (m)(\delta _{s-2}^{-\eta })^m}\exp {\bigg ({\frac{-m\beta _1}{\delta _{s-1}^{-\eta }\alpha _1\rho _n}}\bigg )}\bigg (\sum _{n=0}^{m-1}\bigg (\frac{m}{{\delta _{s-1}^{-\eta }}}\bigg )^n\bigg (\frac{\beta _1}{\alpha _1\rho _n}\bigg )^n\frac{1}{0!(n-0)!}\bigg )\\ &\times \frac{\Gamma (m)}{m^m}\left( \frac{\delta _{s-1}^{-\eta }\delta _{s-2}^{-\eta }}{\frac{\beta _1\alpha _2}{\alpha _1}\delta _{s-2}^{-\eta }+\delta _{s-1}^{-\eta }}\right) ^{m} \\ &-\frac{m^m}{\Gamma (m)(\delta _{s-2}^{-\eta })^m}\left( \frac{\beta _1\alpha _2}{\alpha _1}\right) \exp {\bigg ({\frac{-m\beta _1}{\delta _{s-1}^{-\eta }\alpha _1\rho _n}}\bigg )}\bigg (\sum _{n=1}^{m-1}\bigg (\frac{m}{\delta _{s-1}^{-\eta }}\bigg )^n\bigg (\frac{\beta _1}{\alpha _1\rho _n}\bigg )^{n-1}\frac{1}{1!(n-1)!}\bigg )\\ &\frac{\Gamma (m+1)}{m^{m+1}}\left( \frac{\delta _{s-1}^{-\eta }\delta _{s-2}^{-\eta }}{\frac{\beta _1\alpha _2}{\alpha _1}\delta _{s-2}^{-\eta }+\delta _{s-1}^{-\eta }}\right) ^{m+1} \\ &-\frac{m^m}{\Gamma (m)(\delta _{s-2}^{-\eta })^m}\left( \frac{\beta _1\alpha _2}{\alpha _1}\right) ^2\exp {\bigg ({\frac{-m\beta _1}{\delta _{s-1}^{-\eta }\alpha _1\rho _n}}\bigg )}\bigg (\sum _{n=2}^{m-1}\bigg (\frac{m}{\delta _{s-1}^{-\eta }}\bigg )^n\bigg (\frac{\beta _1}{\alpha _1\rho _n}\bigg )^{n-2}\frac{1}{2!(n-2)!}\bigg )\\ &\frac{\Gamma (m+2)}{m^{m+2}}\left( \frac{\delta _{s-1}^{-\eta }\delta _{s-2}^{-\eta }}{\frac{\beta _1\alpha _2}{\alpha _1}\delta _{s-2}^{-\eta }+\delta _{s-1}^{-\eta }}\right) ^{m+2} \\ &-\frac{m^m}{\Gamma (m)(\delta _{s-2}^{-\eta })^m}\left( \frac{\beta _1\alpha _2}{\alpha _1}\right) ^3\exp {\bigg ({\frac{-m\beta _1}{\delta _{s-1}^{-\eta }\alpha _1\rho _n}}\bigg )}\bigg (\sum _{n=3}^{m-1}\bigg (\frac{m}{\delta _{s-1}^{-\eta }}\bigg )^n\bigg (\frac{\beta _1}{\alpha _1\rho _n}\bigg )^{n-3}\frac{1}{3!(n-3)!}\bigg )\\ &\frac{\Gamma (m+3)}{m^{m+3}}\left( \frac{\delta _{s-1}^{-\eta }\delta _{s-2}^{-\eta }}{\frac{\beta _1\alpha _2}{\alpha _1}\delta _{s-2}^{-\eta }+\delta _{s-1}^{-\eta }}\right) ^{m+3} \\ &...\; -\frac{m^m}{\Gamma (m)(\delta _{s-2}^{-\eta })^m}\left( \frac{\beta _1\alpha _2}{\alpha _1}\right) ^{m-1}\exp {\bigg ({\frac{-m\beta _1}{\delta _{s-1}^{-\eta }\alpha _1\rho _n}}\bigg )}\sum _{n=m-1}^{m-1}\frac{m^n}{n!(\delta _{s-1}^{-\eta })^n}\\ &\frac{\Gamma (2m-1)}{m^{2m-1}}\left( \frac{\delta _{s-1}^{-\eta }\delta _{s-2}^{-\eta }}{\frac{\beta _1\alpha _2}{\alpha _1}\delta _{s-2}^{-\eta }+\delta _{s-1}^{-\eta }}\right) ^{2m-1} \end{aligned} \end{aligned}$$

The multiple summations and Gamma function terms arise from the series expansion of the incomplete Gamma functions. Each term corresponds to a higher-order contribution of interference and fading effects, allowing the final closed form in (38). The closed-form expression for the probability of outage for $\text {UD}_1$ at gNB is obtained by rearranging (37), and the final expression is represented as,

38 $$\begin{aligned} PO_{UD_1}^{UL}= & 1-\left[ \frac{1}{(\delta _{s-2}^{-\eta })^m}{\exp \left( {\frac{-m\beta _1}{\alpha _1\rho _n\delta _{s-1}^{-\eta }}}\right) }\sum _{n=0}^{m-1}\frac{(m+n-1)!}{(m-n)!m^n}\left( \frac{\beta _1\alpha _2}{\alpha _1}\right) ^n\left( \frac{\delta _{s-1}^{-\eta }\delta _{s-2}^{-\eta }}{\frac{\beta _1\alpha _2}{\alpha _1}\delta _{s-2}^{-\eta }+\delta _{s-1}^{-\eta }}\right) ^{m+n} \right. \nonumber \\ & \left. \sum _{k=n}^{m-1}\frac{1}{n!(k-n)!}\left( \frac{m}{\delta _{s-1}^{-\eta }}\right) ^k\left( \frac{\beta _1}{\alpha _1\rho _n}\right) ^{k-n}\right] \end{aligned}$$

Equation (38) is used to calculate the outage probability for $\text {UD}_1$ in the UL-NOMA system. It consists of an exponential term defined in terms of $m,\beta _j$, PA factors, average channel gain, and SNR. Based on the conditions outlined in (29), the outage probability of $\text {UD}_2$ is defined and articulated as,

39 $$\begin{aligned} PO_{UD_2}^{UL}=1-\int _{\Theta _{s-2}^{UL}}^{\infty }{\underbrace{\left\{ {\int ^{\infty }_{\Theta _{s-1}^{UL}} f_{G_{s-1}}(g_{s-1})dg_{s-1}}\right\} }_{F^{UL-2}_{G_{s-1}} (g_{s-1})} f_{G_{s-2}} (g_{s-2})}dg_{s-2} \end{aligned}$$

The expression for ${F^{UL-2}_{G_{s-1}} (g_{s-1})}$ can be represented in terms of the upper incomplete Gamma function. It is expressed as,

40 $$\begin{aligned} {F^{UL-2}_{G_{s-1}} (g_{s-1})} = \frac{\Gamma \bigg (m,{\frac{m\Theta _{s-1}^{UL}}{\delta _{s-1}^{-\eta }}}\bigg )}{\Gamma (m)} \end{aligned}$$

For further derivation, the upper incomplete Gamma function is represented in terms of exponential series, which is generally represented as^38^,

41 $$\begin{aligned} \Gamma (s,t) = (s-1)!\exp (-t)\left( 1+t+\frac{t^2}{2!}+\frac{t^3}{3!}+...\frac{t^{s-1}}{(s-1)!}\right) \end{aligned}$$

where *s* is an integer. The upper incomplete Gamma function in (40) is expanded in terms of exponential series as in (41), and it is expressed as,

42 $$\begin{aligned} {F^{UL-2}_{G_{s-1}} (g_{s-1})}= & \frac{(m-1)!}{\Gamma (m)}\exp {\left( -{\frac{m\Theta _{s-1}^{UL}}{\delta _{s-1}^{-\eta }}}\right) }\bigg (1+{\left( {\frac{m\Theta _{s-1}^{UL}}{\delta _{s-1}^{-\eta }}}\right) }+\frac{1}{2!}{\left( {\frac{m\Theta _{s-1}^{UL}}{\delta _{s-1}^{-\eta }}}\right) }^2+\frac{1}{3!}{\left( {\frac{m\Theta _{s-1}^{UL}}{\delta _{s-1}^{-\eta }}}\right) }^3\nonumber \\ & +...\frac{1}{(m-1)!}{\left( {\frac{m\Theta _{s-1}^{UL}}{\delta _{s-1}^{-\eta }}}\right) }^{m-1}\bigg ) \end{aligned}$$

where *m* is considered as an integer and $\Gamma (m)=(m-1)!$. Replace $\Theta _{s-1}^{UL}$ using (29) results,

43 $$\begin{aligned} \begin{aligned} {F^{UL-2}_{G_{s-1}} (g_{s-1})}&= \exp {\left( -{\frac{m}{\delta _{s-1}^{-\eta }}} \left( {\frac{\beta _1}{\alpha _1\rho _n}+\frac{\beta _1\alpha _2}{\alpha _1}g_{s-2}}\right) \right) } \bigg (1+{\left( {\frac{m}{\delta _{s-1}^{-\eta }}}\right) \left( {\frac{\beta _1}{\alpha _1\rho _n}+\frac{\beta _1\alpha _2}{\alpha _1}g_{s-2}}\right) }\\ &+\frac{1}{2!}{\left( {\frac{m}{\delta _{s-1}^{-\eta }}}\right) }^2\left( {\frac{\beta _1}{\alpha _1\rho _n}+\frac{\beta _1\alpha _2}{\alpha _1}g_{s-2}}\right) ^2 \\ &+\frac{1}{3!}{\left( {\frac{m}{\delta _{s-1}^{-\eta }}}\right) }^3\left( {\frac{\beta _1}{\alpha _1\rho _n}+\frac{\beta _1\alpha _2}{\alpha _1}g_{s-2}}\right) ^3\\ &+...\frac{1}{(m-1)!}{\left( {\frac{m}{\delta _{s-1}^{-\eta }}}\right) }^{m-1}\left( {\frac{\beta _1}{\alpha _1\rho _n}+\frac{\beta _1\alpha _2}{\alpha _1}g_{s-2}}\right) ^{m-1}\bigg ) \end{aligned} \end{aligned}$$

Applying binomial expansion and arranging different powers of $g_{s-1}$ together, results,

44 $$\begin{aligned} \begin{aligned} {F^{UL-2}_{G_{s-1}} (g_{s-1})} =&\exp {\left( -{\frac{m}{\delta _{s-1}^{-\eta }}} \left( {\frac{\beta _1}{\alpha _1\rho _n} +\frac{\beta _1\alpha _2}{\alpha _1}g_{s-2}}\right) \right) } \bigg (\sum _{n=0}^{m-1}\frac{1}{n!}\left( {\frac{m}{\delta _{s-1}^{-\eta }}}\right) ^n \bigg (\frac{\beta _1}{\alpha _1\rho _n}\bigg )^n\frac{n!}{0!(n-0)!} \\ &+\left( \frac{\beta _1\alpha _2}{\alpha _1}\right) g_{s-2}\sum _{n=1}^{m-1} \left( \frac{1}{n!}\right) \left( {\frac{m}{\delta _{s-1}^{-\eta }}}\right) ^n\bigg (\frac{\beta _1}{\alpha _1\rho _n}\bigg )^{n-1}\frac{n!}{1!(n-1)!} \\ &+\left( \frac{\beta _1\alpha _2}{\alpha _1}\right) ^2g_{s-2}^2\sum _{n=2}^{m-1}\left( \frac{1}{n!}\right) \left( {\frac{m}{\delta _{s-1}^{-\eta }}}\right) ^n\bigg (\frac{\beta _1}{\alpha _1\rho _n}\bigg )^{n-2}\frac{n!}{2!(n-2)!} \\ &+\left( \frac{\beta _1\alpha _2}{\alpha _1}\right) ^3g_{s-2}^3\sum _{n=3}^{m-1}\left( \frac{1}{n!}\right) \left( {\frac{m}{\delta _{s-1}^{-\eta }}}\right) ^n\bigg (\frac{\beta _1}{\alpha _1\rho _n}\bigg )^{n-3}\frac{n!}{3!(n-3)!} \\ &...\; +\left( \frac{\beta _1\alpha _2}{\alpha _1}\right) ^{m-1}g_{s-2}^{m-1}\sum _{n=m-1}^{m-1}\left( \frac{1}{n!}\right) \left( {\frac{m}{\delta _{s-1}^{-\eta }}}\right) ^n\\ &\bigg (\frac{\beta _1}{\alpha _1\rho _n}\bigg )^{n-(m-1)}\frac{n!}{(m-1)!(n-(m-1))!} \bigg ) \end{aligned} \end{aligned}$$

Substituting (1) for $j=2$, and (44) in (39). Expanding the terms and distributing the integral over each individual term results,

45 $$\begin{aligned} \begin{aligned} PO_{UD_2}^{UL}&=1-\frac{m^m}{\Gamma (m)(\delta _{s-2}^{-\eta })^m}\exp {\bigg ({\frac{-m\beta _1}{\delta _{s-1}^{-\eta }\alpha _1\rho _n}}\bigg )}\bigg (\sum _{n=0}^{m-1}\bigg (\frac{m}{{\delta _{s-1}^{-\eta }}}\bigg )^n\bigg (\frac{\beta _1}{\alpha _1\rho _n}\bigg )^n\frac{1}{0!(n-0)!}\bigg ) \\ &\int _{\Theta _{s-2}^{UL}}^{\infty } g_{s-2}^{m-1}\exp \left( {\left( \frac{-m}{\delta _{s-2}^{-\eta }}-{\frac{m\beta _1\alpha _2}{\delta _{s-1}^{-\eta }\alpha _1}}\right) g_{s-2}}\right) dg_{s-2} \\ &-\frac{m^m}{\Gamma (m)(\delta _{s-2}^{-\eta })^m}\left( \frac{\beta _1\alpha _2}{\alpha _1}\right) \exp {\bigg ({\frac{-m\beta _1}{\delta _{s-1}^{-\eta }\alpha _1\rho _n}}\bigg )}\bigg (\sum _{n=1}^{m-1}\bigg (\frac{m}{\delta _{s-1}^{-\eta }}\bigg )^n\bigg (\frac{\beta _1}{\alpha _1\rho _n}\bigg )^{n-1}\frac{1}{1!(n-1)!}\bigg )\\ &\int _{\Theta _{s-2}^{UL}}^{\infty }g_{s-2}^{m}\exp \left( {\left( \frac{-m}{\delta _{s-2}^{-\eta }}-{\frac{m\beta _1\alpha _2}{\delta _{s-1}^{-\eta }\alpha _1}}\right) g_{s-2}}\right) dg_{s-2} \\ &-\frac{m^m}{\Gamma (m)(\delta _{s-2}^{-\eta })^m}\left( \frac{\beta _1\alpha _2}{\alpha _1}\right) ^2\exp {\bigg ({\frac{-m\beta _1}{\delta _{s-1}^{-\eta }\alpha _1\rho _n}}\bigg )}\bigg (\sum _{n=2}^{m-1}\bigg (\frac{m}{\delta _{s-1}^{-\eta }}\bigg )^n\bigg (\frac{\beta _1}{\alpha _1\rho _n}\bigg )^{n-2}\frac{1}{2!(n-2)!}\bigg )\\ &\int _{\Theta _{s-2}^{UL}}^{\infty }g_{s-2}^{m+1}\exp \left( {\left( \frac{-m}{\delta _{s-2}^{-\eta }}-{\frac{m\beta _1\alpha _2}{\delta _{s-1}^{-\eta }\alpha _1}}\right) g_{s-2}}\right) dg_{s-2} \\ &-\frac{m^m}{\Gamma (m)(\delta _{s-2}^{-\eta })^m}\left( \frac{\beta _1\alpha _2}{\alpha _1}\right) ^3\exp {\bigg ({\frac{-m\beta _1}{\delta _{s-1}^{-\eta }\alpha _1\rho _n}}\bigg )}\bigg (\sum _{n=3}^{m-1}\bigg (\frac{m}{\delta _{s-1}^{-\eta }}\bigg )^n\bigg (\frac{\beta _1}{\alpha _1\rho _n}\bigg )^{n-3}\frac{1}{3!(n-3)!}\bigg )\\ &\int _{\Theta _{s-2}^{UL}}^{\infty }g_{s-2}^{m+2}\exp \left( {\left( \frac{-m}{\delta _{s-2}^{-\eta }}-{\frac{m\beta _1\alpha _2}{\delta _{s-1}^{-\eta }\alpha _1}}\right) g_{s-2}}\right) dg_{s-2} \\ &...\; -\frac{m^m}{\Gamma (m)(\delta _{s-2}^{-\eta })^m}\left( \frac{\beta _1\alpha _2}{\alpha _1}\right) ^{m-1}\exp {\bigg ({\frac{-m\beta _1}{\delta _{s-1}^{-\eta }\alpha _1\rho _n}}\bigg )}\sum _{n=m-1}^{m-1}\frac{m^n}{n!(\delta _{s-1}^{-\eta })^n}\\ &\int _{\Theta _{s-2}^{UL}}^{\infty }g_{s-2}^{2(m-1)}\exp \left( {\left( \frac{-m}{\delta _{s-2}^{-\eta }}-{\frac{m\beta _1\alpha _2}{\delta _{s-1}^{-\eta }\alpha _1}}\right) g_{s-2}}\right) dg_{s-2} \end{aligned} \end{aligned}$$

By performing the calculations and incorporating the integration of (45), the resulting equation is expressed as,

46 $$\begin{aligned} \begin{aligned} PO_{UD_2}^{UL}&=1-\frac{m^m\exp {\bigg ({\frac{-m\beta _1}{\delta _{s-1}^{-\eta }\alpha _1\rho _n}}\bigg )}}{\Gamma (m)(\delta _{s-2}^{-\eta })^m}\bigg (\sum _{n=0}^{m-1}\bigg (\frac{m}{{\delta _{s-1}^{-\eta }}}\bigg )^n\bigg (\frac{\beta _1}{\alpha _1\rho _n}\bigg )^n\frac{1}{0!(n-0)!}\bigg )\\ &\frac{{\Gamma \bigg (m,m\left( \frac{\frac{\beta _1\alpha _2}{\alpha _1}\delta _{s-2}^{-\eta }+\delta _{s-1}^{-\eta }}{\delta _{s-1}^{-\eta }\delta _{s-2}^{-\eta }}\right) \Theta _{s-2}^{UL}\bigg )}}{m^m\left( \frac{\frac{\beta _1\alpha _2}{\alpha _1}\delta _{s-2}^{-\eta }+\delta _{s-1}^{-\eta }}{\delta _{s-1}^{-\eta }\delta _{s-2}^{-\eta }}\right) ^m} \\ &-\frac{m^m\exp {\bigg ({\frac{-m\beta _1}{\delta _{s-1}^{-\eta }\alpha _1\rho _n}}\bigg )}}{\Gamma (m)(\delta _{s-2}^{-\eta })^m}\left( \frac{\beta _1\alpha _2}{\alpha _1}\right) \bigg (\sum _{n=1}^{m-1}\bigg (\frac{m}{\delta _{s-1}^{-\eta }}\bigg )^n\bigg (\frac{\beta _1}{\alpha _1\rho _n}\bigg )^{n-1}\frac{1}{1!(n-1)!}\bigg ) \\ &\frac{{\Gamma \bigg ((m+1),m\left( \frac{\frac{\beta _1\alpha _2}{\alpha _1}\delta _{s-2}^{-\eta }+\delta _{s-1}^{-\eta }}{\delta _{s-1}^{-\eta }\delta _{s-2}^{-\eta }}\right) \Theta _{s-2}^{UL}\bigg )}}{m^{m+1}\left( \frac{\frac{\beta _1\alpha _2}{\alpha _1}\delta _{s-2}^{-\eta }+\delta _{s-1}^{-\eta }}{\delta _{s-1}^{-\eta }\delta _{s-2}^{-\eta }}\right) ^{m+1}} \\ &-\frac{m^m\exp {\bigg ({\frac{-m\beta _1}{\delta _{s-1}^{-\eta }\alpha _1\rho _n}}\bigg )}}{\Gamma (m)(\delta _{s-2}^{-\eta })^m}\left( \frac{\beta _1\alpha _2}{\alpha _1}\right) ^2\bigg (\sum _{n=2}^{m-1}\bigg (\frac{m}{\delta _{s-1}^{-\eta }}\bigg )^n\bigg (\frac{\beta _1}{\alpha _1\rho _n}\bigg )^{n-2}\frac{1}{2!(n-2)!}\bigg ) \\ &\frac{{\Gamma \bigg ((m+2),m\left( \frac{\frac{\beta _1\alpha _2}{\alpha _1}\delta _{s-2}^{-\eta }+\delta _{s-1}^{-\eta }}{\delta _{s-1}^{-\eta }\delta _{s-2}^{-\eta }}\right) \Theta _{s-2}^{UL}\bigg )}}{m^{m+2}\left( \frac{\frac{\beta _1\alpha _2}{\alpha _1}\delta _{s-2}^{-\eta }+\delta _{s-1}^{-\eta }}{\delta _{s-1}^{-\eta }\delta _{s-2}^{-\eta }}\right) ^{m+2}} \\ &-\frac{m^m\exp {\bigg ({\frac{-m\beta _1}{\delta _{s-1}^{-\eta }\alpha _1\rho _n}}\bigg )}}{\Gamma (m)(\delta _{s-2}^{-\eta })^m}\left( \frac{\beta _1\alpha _2}{\alpha _1}\right) ^3\bigg (\sum _{n=3}^{m-1}\bigg (\frac{m}{\delta _{s-1}^{-\eta }}\bigg )^n\bigg (\frac{\beta _1}{\alpha _1\rho _n}\bigg )^{n-3}\frac{1}{3!(n-3)!}\bigg ) \\ &\frac{{\Gamma \bigg ((m+3),m\left( \frac{\frac{\beta _1\alpha _2}{\alpha _1}\delta _{s-2}^{-\eta }+\delta _{s-1}^{-\eta }}{\delta _{s-1}^{-\eta }\delta _{s-2}^{-\eta }}\right) \Theta _{s-2}^{UL}\bigg )}}{m^{m+3}\left( \frac{\frac{\beta _1\alpha _2}{\alpha _1}\delta _{s-2}^{-\eta }+\delta _{s-1}^{-\eta }}{\delta _{s-1}^{-\eta }\delta _{s-2}^{-\eta }}\right) ^{m+3}} \\ &...\; -\frac{m^m\exp {\bigg ({\frac{-m\beta _1}{\delta _{s-1}^{-\eta }\alpha _1\rho _n}}\bigg )}}{\Gamma (m)(\delta _{s-2}^{-\eta })^m}\left( \frac{\beta _1\alpha _2}{\alpha _1}\right) ^{m-1}\sum _{n=m-1}^{m-1}\frac{m^n}{n!(\delta _{s-1}^{-\eta })^n} \\ &\frac{{\Gamma \bigg ((2m-1),m\left( \frac{\frac{\beta _1\alpha _2}{\alpha _1}\delta _{s-2}^{-\eta }+\delta _{s-1}^{-\eta }}{\delta _{s-1}^{-\eta }\delta _{s-2}^{-\eta }}\right) \Theta _{s-2}^{UL}\bigg )}}{m^{2m-1}\left( \frac{\frac{\beta _1\alpha _2}{\alpha _1}\delta _{s-2}^{-\eta }+\delta _{s-1}^{-\eta }}{\delta _{s-1}^{-\eta }\delta _{s-2}^{-\eta }}\right) ^{2m-1}} \end{aligned} \end{aligned}$$

The closed-form expression for the probability of outage for $\text {UD}_2$ at gNB is obtained by rearranging (46), and the final expression is represented as,

47 $$\begin{aligned} \begin{aligned} PO_{UD_2}^{UL}=1-&\bigg [\frac{{\exp \left( {\frac{-m\beta _1}{\alpha _1\rho _n\delta _{s-1}^{-\eta }}}\right) }}{\Gamma (m)(\delta _{s-2}^{-\eta })^m}\sum _{n=0}^{m-1}{{\Gamma \bigg ((m+n),m\left( \frac{\frac{\beta _1\alpha _2}{\alpha _1}\delta _{s-2}^{-\eta }+\delta _{s-1}^{-\eta }}{\delta _{s-1}^{-\eta }\delta _{s-2}^{-\eta }}\right) \Theta _{s-2}^{UL}\bigg )}}\left( \frac{\beta _1\alpha _2}{m\alpha _1}\right) ^n\\ &\left( \frac{\delta _{s-1}^{-\eta }\delta _{s-2}^{-\eta }}{\frac{\beta _1\alpha _2}{\alpha _1}\delta _{s-2}^{-\eta }+\delta _{s-1}^{-\eta }}\right) ^{m+n} \\ &\times \sum _{k=n}^{m-1}\frac{1}{n!(k-n)!}\left( \frac{m}{\delta _{s-1}^{-\eta }}\right) ^k\left( \frac{\beta _1}{\alpha _1\rho _n}\right) ^{k-n}\bigg ] \end{aligned} \end{aligned}$$

Equation (47) is used to calculate the outage probability for $\text {UD}_2$ in the UL-NOMA system. It consists of an exponential term and upper incomplete Gamma function defined in terms of $m,\beta _j$, PA factors, and average channel gain. The condition for the probability of outage for $\text {UD}_1$ in terms of $g_{s-1}$ is derived in (28), which is the upper limit of integration operation of the outage equation and is represented as, $g_{s-1}<{\frac{\beta _1}{\alpha _1\rho _n}+\frac{\beta _1\alpha _2}{\alpha _1}g_{s-2}}$, where $\rho _n$ represents SNR. For a higher range of $\rho _n$, the first term in the condition will be neglected compared to the second term, and the condition will change and it is expressed as,

48 $$\begin{aligned} g_{s-1}<\underbrace{\frac{\beta _1\alpha _2}{\alpha _1}g_{s-2}}_{\Theta _{s-1}^{UL}} \end{aligned}$$

It can be observed that the limits of integration no longer depend on $\rho _n$. Hence, the outage for $\text {UD}_1$ reaches a saturation level. In UL-NOMA, this saturation point is termed the outage floor. The outage floor is a constant value below which the outage will not fall, even if the SNR increases. For $\text {UD}_1$, this can be calculated by substituting the derived limit value of (48) into (30).

The condition for the probability of outage for $\text {UD}_2$ includes two conditions derived in (29), which is the upper limit of integration operation of the outage equation and is represented as, $g_{s-1}<{\frac{\beta _1}{\alpha _1\rho _n}+\frac{\beta _1\alpha _2}{\alpha _1}g_{s-2}},\; g_{s-2}<{\frac{\beta _2}{\alpha _2\rho _n}}$. For a higher range of $\rho _n$, the conditions changes as,

49 $$\begin{aligned} g_{s-1}<\underbrace{\frac{\beta _1\alpha _2}{\alpha _1}g_{s-2}}_{\Theta _{s-1}^{UL}},\; g_{s-2}\approx \underbrace{{0}}_{\Theta _{s-2}^{UL}} \end{aligned}$$

It can be observed that the limits of integration no longer depend on $\rho _n$. Hence, the outage for $\text {UD}_2$ reaches a saturation level, which is the outage floor for $\text {UD}_2$. For $\text {UD}_2$, this can be calculated using the derived limit value of (49) into (39). The outage floor in UL-NOMA systems represents the threshold at which further increases in the SNR will not reduce the outage probability, demonstrating a performance limitation in real-time scenarios. The existence of an outage floor is essential for system designers, as it emphasizes the highest level of reliability that can be attained. This facilitates efficient power distribution and highlights the need for refined methods to enhance system performance. It also differentiates the best possible performance from what can actually be achieved in practical situations.

### Outage probability analysis in UL-NOMA with HI and *i*SIC

In UL-NOMA, the data outage condition for $\text {UD}_1$ at gNB under HI and *i*SIC is denoted as $R_{s-1}^{im-UL}<\tilde{\beta }_1$. The condition for the probability of outage in terms of $g_{s-1}$ is determined by substituting (16) for $j=1$ into the condition, and it results,

50 $$\begin{aligned} g_{s-1}<\underbrace{\frac{\beta _1}{(\alpha _1-\alpha _1\beta _1\lambda ^2)\rho _n}+\frac{\beta _1\alpha _2(1+\lambda ^2)}{(\alpha _1-\alpha _1\beta _1\lambda ^2)}g_{s-2}}_{\Theta _{s-1}^{im-UL}} \end{aligned}$$

where ${\Theta _{s-1}^{im-UL}}$ is defined in terms of $g_{s-2}$, which varies from zero to infinity. The gNB performs SIC to decode $\text {UD}_2$’s data. Hence the condition for outage for $\text {UD}_2$ considers the residual part of $\text {UD}_1$’s data, determined by the parameter $\varepsilon$. The conditions are denoted as $R_{s-1}^{im-UL}<\tilde{\beta }_1$ and $R_{s-2}^{im-UL}<\tilde{\beta }_2$. When either of these conditions is satisfied, $\text {UD}_2$ will be in outage. The corresponding ranges for $g_{s-1}$ and $g_{s-2}$ is determined by substituting (16) into the conditions, and it results,

51 $$\begin{aligned} g_{s-1}<\underbrace{\frac{\beta _1}{(\alpha _1-\alpha _1\beta _1\lambda ^2)\rho _n}+\frac{\beta _1\alpha _2(1+\lambda ^2)}{(\alpha _1-\alpha _1\beta _1\lambda ^2)}g_{s-2}}_{\Theta _{s-1}^{im-UL}}, \; g_{s-2}<\underbrace{\frac{\beta _2}{(\alpha _2-\beta _2 \alpha _2 \lambda ^2)\rho _n}+\frac{\varepsilon \beta _2\alpha _1(1+\lambda ^2)}{(\alpha _2-\beta _2\alpha _2 \lambda ^2)}g_{s-1}}_{\Theta _{s-2}^{im-UL}} \end{aligned}$$

In UL-NOMA, the gNB directly decodes $\text {UD}_1$’s data, allowing the information related to $\text {UD}_1$ to remain unaffected by *i*SIC. Based on the conditions outlined in (50), the outage probability of $\text {UD}_1$ with HI is defined and articulated as,

52 $$\begin{aligned} PO_{UD_1}^{im-UL}=\int _{0}^{\infty }{{\left\{ {\int ^{{\Theta _{s-1}^{im-UL}}}_{0} f_{G_{s-1}}(g_{s-1})dg_{s-1}}\right\} } f_{G_{s-2}} (g_{s-2})}dg_{s-2} \end{aligned}$$

The expression for $PO_{UD_1}^{im-UL}$ is similar to (30) with changes in the integration limit. Hence, the result is obtained by updating (38). The probability of outage for $\text {UD}_1$ in UL-NOMA with HI is expressed as,

53 $$\begin{aligned} \begin{aligned} PO_{UD_1}^{im-UL}&= 1-\bigg [\frac{1}{(\delta _{s-2}^{-\eta })^m}{\exp \left( \frac{m\beta _1}{(\alpha _1-\alpha _1\beta _1\lambda ^2)\rho _n\delta _{s-1}^{-\eta }}\right) }\\ &\sum _{n=0}^{m-1}\frac{(m+n-1)!}{(m-n)!m^n}\left( \frac{\beta _1\alpha _2(1+\lambda ^2)}{(\alpha _1-\alpha _1\beta _1\lambda ^2)}\right) ^n\left( \frac{\delta _{s-1}^{-\eta }\delta _{s-2}^{-\eta }}{\frac{\beta _1\alpha _2(1+\lambda ^2)}{(\alpha _1-\alpha _1\beta _1\lambda ^2)}\delta _{s-2}^{-\eta }+\delta _{s-1}^{-\eta }}\right) ^{m+n} \\ &\times \sum _{k=n}^{m-1}\frac{1}{n!(k-n)!}\left( \frac{m}{\delta _{s-1}^{-\eta }}\right) ^k\left( \frac{\beta _1}{(\alpha _1-\alpha _1\beta _1\lambda ^2)\rho _n}\right) ^{k-n}\bigg ] \end{aligned} \end{aligned}$$

The conditions for $\text {UD}_2$’s outage probability are defined in (51). It consists of two specific ranges, defined in terms of $g_{s-1}$ and $g_{s-2}$. The equation defining the range associated with $g_{s-1}$ has already been specified in (52), and the corresponding result in (53) is denoted as $PO_{UD_1}^{im-UL}$. Let the equation associated with the range of $g_{s-2}$ is represented as,

54 $$\begin{aligned} \tilde{PO}_{UD_2}^{im-UL}=\int _{0}^{\infty }{{\left\{ {\int ^{{\Theta _{s-2}^{im-UL}}}_{0} f_{G_{s-2}}(g_{s-2})dg_{s-2}}\right\} } f_{G_{s-1}} (g_{s-1})}dg_{s-1} \end{aligned}$$

The expression for $\tilde{PO}_{UD_2}^{im-UL}$ is similar to (52) with changes in the integration limit and the PDF. Hence, the result is obtained by updating (53) with corresponding changes, and it is expressed as,

55 $$\begin{aligned} \begin{aligned} \tilde{PO}_{UD_2}^{im-UL}&= 1-\bigg [\frac{1}{(\delta _{s-1}^{-\eta })^m}{\exp \left( \frac{m\beta _2}{(\alpha _2-\beta _2 \alpha _2 \lambda ^2)\rho _n\delta _{s-2}^{-\eta }}\right) }\\ &\sum _{n=0}^{m-1}\frac{(m+n-1)!}{(m-n)!m^n}\left( \frac{\varepsilon \beta _2\alpha _1(1+\lambda ^2)}{(\alpha _2-\beta _2\alpha _2 \lambda ^2)}\right) ^n\left( \frac{\delta _{s-1}^{-\eta }\delta _{s-2}^{-\eta }}{\frac{\varepsilon \beta _2\alpha _1(1+\lambda ^2)}{(\alpha _2-\beta _2\alpha _2 \lambda ^2)}\delta _{s-2}^{-\eta }+\delta _{s-1}^{-\eta }}\right) ^{m+n} \\ &\times \sum _{k=n}^{m-1}\frac{1}{n!(k-n)!}\left( \frac{m}{\delta _{s-2}^{-\eta }}\right) ^k\left( \frac{\beta _2}{(\alpha _2-\beta _2 \alpha _2 \lambda ^2)\rho _n}\right) ^{k-n}\bigg ] \end{aligned} \end{aligned}$$

The derived range for $g_{s-1}$ in (51) depends on $g_{s-2}$ and vice versa.

In UL-NOMA, gNB performs SIC to obtain the information for $\text {UD}_2$. Hence, the probability of outage for $\text {UD}_2$ depends on the outage for $\text {UD}_1$, as represented in (53), and also depends on the outage for $\text {UD}_2$ data without considering SIC as represented in (55). To accurately derive the total outage probability of $\text {UD}_2$, it is necessary to consider both the individual outage probabilities of $\text {UD}_1$ and $\text {UD}_2$ while eliminating the overlap where both users are simultaneously in outage^37^. This is achieved by applying the inclusion-exclusion principle and the corresponding outage probability for $\text {UD}_2$ with HI and *i*SIC is expressed as,

56 $$\begin{aligned} \begin{aligned} {PO}_{UD_2}^{im-UL} = \tilde{PO}_{UD_2}^{im-UL} + PO_{UD_1}^{im-UL} - (\tilde{PO}_{UD_2}^{im-UL}PO_{UD_1}^{im-UL}) \end{aligned} \end{aligned}$$

Here, the third term represents the overlapping region. When the dependence between events $\tilde{PO}_{UD_2}^{im-UL}$ and $PO_{UD_1}^{im-UL}$ is minimum, indicating the coupling term between the variables is very small (i.e., $\frac{\beta \alpha _2}{\alpha _1}$, or parameters such as $\varepsilon$ and/or $\lambda ^2$ are close to zero, the dependence of one event on the other becomes minimal). In such cases, $\tilde{PO}_{UD_2}^{im-UL}$ and $PO_{UD_1}^{im-UL}$ behave nearly as independent events, and the joint probability can be approximated by the product of their individual probabilities, i.e., $\tilde{PO}_{UD_2}^{im-UL}\cap PO_{UD_1}^{im-UL}\approx (\tilde{PO}_{UD_2}^{im-UL}PO_{UD_1}^{im-UL})$. This simplification is incorporated into the equation without a significant loss of accuracy.

## Multi-user NOMA

In NOMA-based systems, an increase in the number of users typically leads to a gradual decline in the SSE. It also introduces high complexity in the SIC process and leads to error propagation. However, these challenges can be alleviated by implementing user pairing strategies. In this section, a hybrid form of NOMA and OMA is discussed to enhance the SSE in a Nakagami-*m* distributed multi-user wireless system, termed HNOMA. The HNOMA system considers time division multiple access (TDMA) as the OMA method, along with NOMA. This method leverages the simplicity in the design of the OMA scheme while attaining the massive connectivity provided by NOMA systems. The users are divided into distinct pairs, with each pair comprising a user with a strong channel and another one with a weak channel condition. In the TDMA-based system, the available time resources are divided based on the number of active user pairs, enabling efficient time slot allocation for each pair. Let $T_i$ represent the time slot assigned to the *i*th user pair, during which the NOMA composite signals will be transmitted and received. In DL-NOMA, the gNB transmits the composite signal for the *i*th pair at the time of $T_i$. In UL-NOMA, the users of the *i*th pair transmit their signal to the gNB at the time of $T_i$.

This paper focuses on three different user pairing methodologies to mitigate the complexity associated with multi-user scenarios. For the explanation of HNOMA the number of users considered is $M=4$. Let $\text {UD}_j$ is located at a distance of $\delta _{s-j}$, where $\delta _{s-1}<\delta _{s-2}<\delta _{s-3}<\delta _{s-4}$, that is, $E\{G_{s-1}\}> E\{G_{s-2}\}>E\{G_{s-3}\}> E\{G_{s-4}\}$. The PA for $\text {UD}_j$ ensures fairness. The users are split into two groups, each consisting of a user pair. According to the formation of the groups, the pairing methods are categorized as follows: spatial proximity (SP) pairing, strong-weak (SW) pairing, index-based (IB) pairing, and random combination (RC) pairing.

**Fig. 3:**
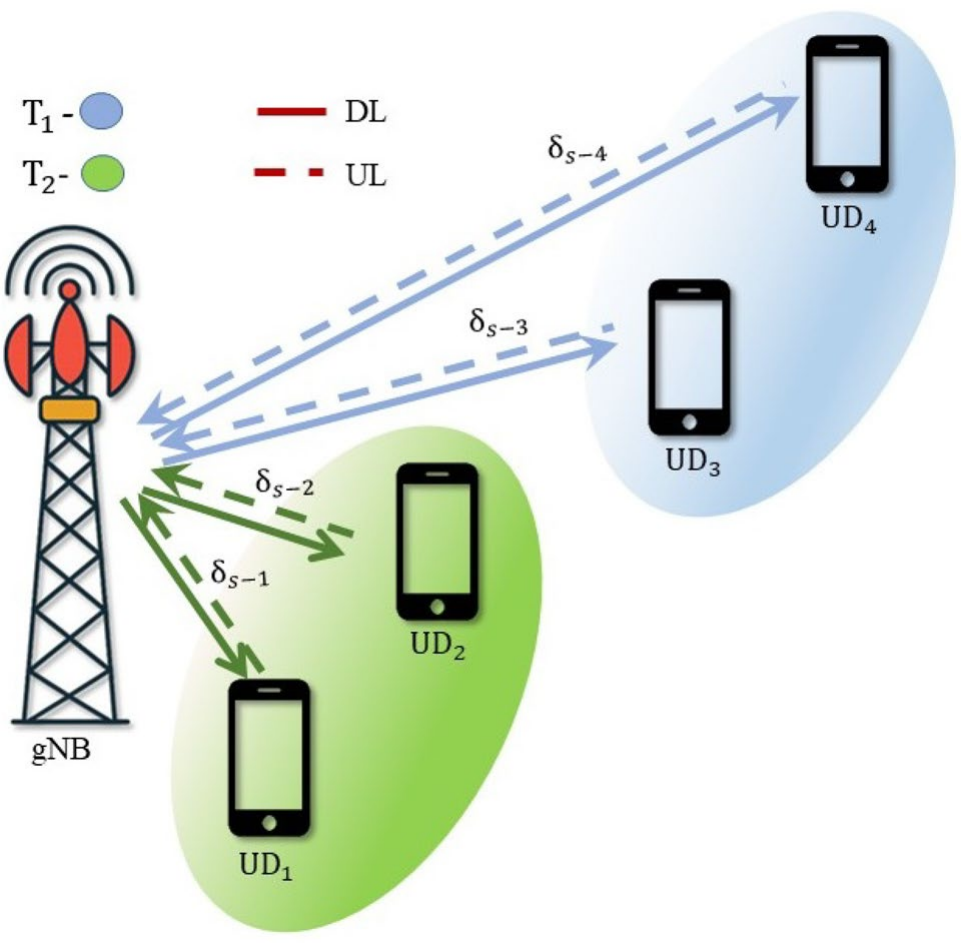
SP pairing.

**Fig. 4:**
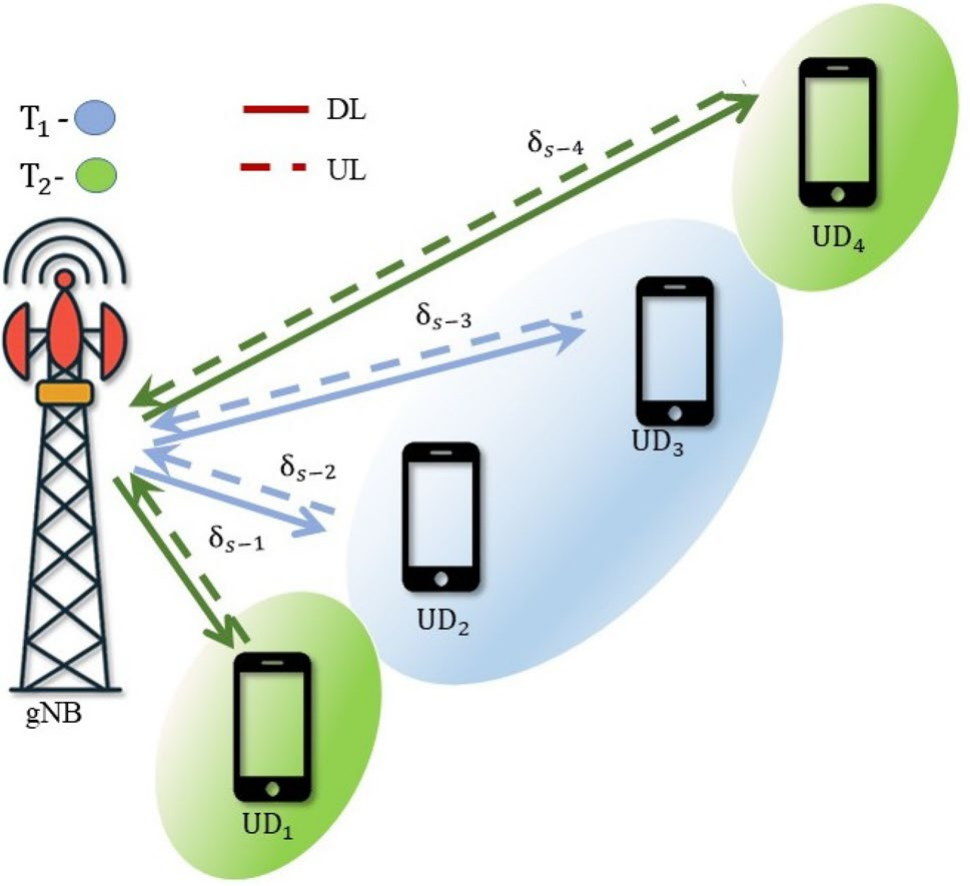
SW pairing.

**Fig. 5:**
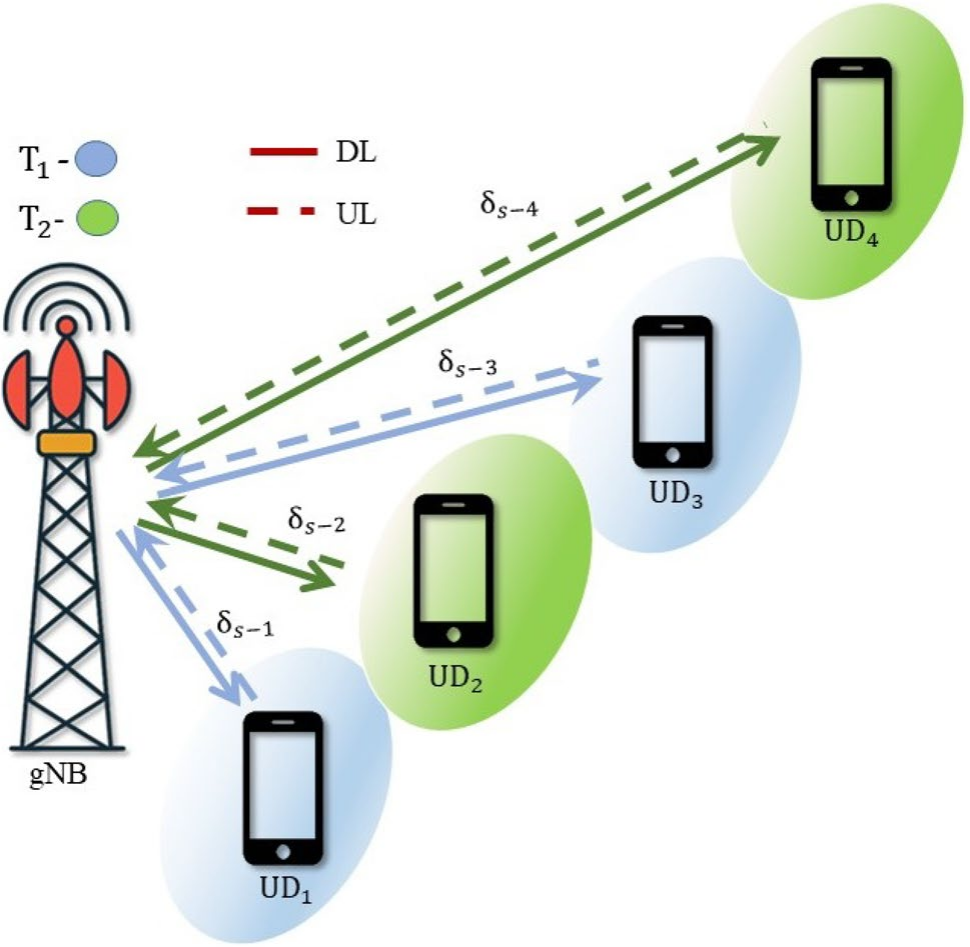
IB pairing.

**Fig. 6:**
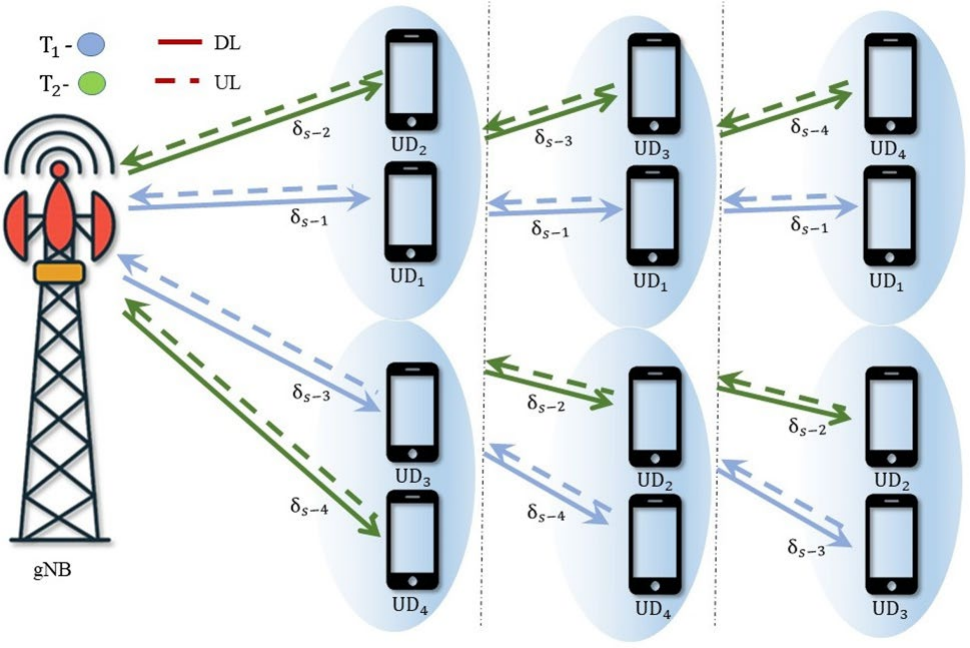
RC pairing.

- *SP pairing* In SP pairing, $\text {UD}_j$ located in close proximity are grouped together, as illustrated in Fig. 3. Here, $\text {UD}_1$ and $\text {UD}_2$ are considered as one pair in which $\text {UD}_1$ has strong channel conditions compared to $\text {UD}_2$. $\text {UD}_3$ and $\text {UD}_4$ are considered as other pair in which $\text {UD}_3$ has strong channel conditions compared to $\text {UD}_4$. The time resource is divided into two slots, $T_1$ and $T_2$, with each slot allocated to a pair. This scheme has a high possibility of having similar channel characteristics for the pair of users. In SP pairing, when the $\text {UD}_j$ within a pair are equidistant from the gNB, it does not leverage the advantages of NOMA. The complexity of this approach is determined by the necessity to sort $\text {UD}_j$ based on their channel gain and select the corresponding pairs, resulting in a computational complexity of $O(M\log {M})$^7^.

- *SW pairing* In SW pairing, the user at the maximum distance from the gNB is paired with the user at the minimum distance. From the remaining users, the identical criteria are used to form the next pairs. The SW pairing scheme is illustrated in Fig. 4. Here, $\text {UD}_1$ is paired with $\text {UD}_4$ and $\text {UD}_2$ is paired with $\text {UD}_3$. In SW pairing the distance maintained between users is maximal when compared to other approaches. This method, like SP pairing, includes sorting and selection of $\text {UD}_j$, resulting in a computational complexity of $O(M\log {M})$^7^.

- *IB pairing* In IB pairing, $\text {UD}_j$ are organized into pairs based on their indices. Specifically, users with odd indices are paired together, while users with even indices form a separate pair. Thus, $\text {UD}_1$ and $\text {UD}_3$ are considered as a pair and $\text {UD}_2$ and $\text {UD}_4$ are considered another pair, as illustrated in Fig. 5. The user indexing is performed according to the channel gain. The complexity of this approach is determined by the user’s indexing process and selection of pairs, resulting in a computational complexity of $O(M\log {M})$^7^.

- *RC pairing* RC pairing considers every combination of the users. Each pair, one user with strong and one with poor channel conditions, is considered. The possible combinations of the user groups consist of the following pairs: ($\text {UD}_1$, $\text {UD}_2$ ) and ($\text {UD}_3$, $\text {UD}_4$), or ($\text {UD}_1$, $\text {UD}_3$) and ($\text {UD}_2$, $\text {UD}_4$), or ($\text {UD}_1$, $\text {UD}_4$) and ($\text {UD}_2$, $\text {UD}_3$). The RC pairing scheme is illustrated in Fig. 6. The information is transmitted or received by any of the listed combinations of $\text {UD}_j$ at random, where each pair will obtain its data during the corresponding $T_i$. The RC pairing has the computational complexity of *O*(*M*)^7^.

### SSE of DL-HNOMA

In this section, the SSE analysis for the DL-HNOMA is performed. Consider the SP pairing, where $\text {UD}_1$ is paired with $\text {UD}_2$, and $\text {UD}_3$ is paired with $\text {UD}_4$. In the initial pair, $\text {UD}_1$ is experiencing good channel conditions, whereas $\text {UD}_2$ has poor channel conditions. Hence, $\text {UD}_1$ performs SIC to decode the data, while $\text {UD}_2$ decodes the data directly. Similarly, in the second pair, $\text {UD}_3$ performs SIC to decode the data, and $\text {UD}_4$ decodes the data directly. The TDMA-based system divides the time resource into equal sections according to the number of pairs. Hence, the individual user’s SE will be reduced by a factor of 1/2, and the corresponding SSE is expressed as,

57 $$\begin{aligned} S_{HNOMA}^{DL-SP}= & \bigg [\frac{1}{2}\log _2(1+g_{s-1} \alpha _1 \rho _n)+\frac{1}{2}\log _2\bigg (1+\frac{g_{s-2} \alpha _2 \rho _n}{1+g_{s-1}\alpha _1 \rho _n }\ \bigg )\bigg ]\nonumber \\ & +\bigg [\frac{1}{2}\log _2(1+g_{s-3} \alpha _3 \rho _n)+\frac{1}{2}\log _2\bigg (1+\frac{g_{s-4} \alpha _4 \rho _n}{1+g_{s-3}\alpha _3 \rho _n }\bigg ) \bigg ] \end{aligned}$$

Similarly, for SW pairing, $\text {UD}_1$ and $\text {UD}_2$ perform SIC to decode the data, while $\text {UD}_3$ and $\text {UD}_4$ decodes the data directly. Hence, the SSE for SW pairing is expressed as,

58 $$\begin{aligned} S_{HNOMA}^{DL-SW}= & \bigg [\frac{1}{2}\log _2(1+g_{s-1} \alpha _1 \rho _n)+\frac{1}{2}\log _2\bigg (1+\frac{g_{s-4} \alpha _4 \rho _n}{1+g_{s-1}\alpha _1 \rho _n }\bigg )\bigg ]\nonumber \\ & +\bigg [\frac{1}{2}\log _2(1+g_{s-2} \alpha _2 \rho _n)+\frac{1}{2}\log _2\bigg (1+\frac{g_{s-3} \alpha _3 \rho _n}{1+g_{s-2}\alpha _2 \rho _n }\bigg )\bigg ] \end{aligned}$$

Similarly, for IB pairing, $\text {UD}_1$ and $\text {UD}_2$ perform SIC to decode the data, while $\text {UD}_3$ and $\text {UD}_4$ decodes the data directly. Hence, the SSE for IB pairing is expressed as,

59 $$\begin{aligned} S_{HNOMA}^{DL-IB}= & \bigg [\frac{1}{2}\log _2(1+g_{s-1} \alpha _1 \rho _n)+\frac{1}{2}\log _2\bigg (1+\frac{g_{s-3} \alpha _3 \rho _n}{1+g_{s-1}\alpha _1 \rho _n } \bigg )\bigg ]\nonumber \\ & +\bigg [\frac{1}{2}\log _2(1+g_{s-2} \alpha _2 \rho _n)+\frac{1}{2}\log _2\bigg (1+\frac{g_{s-4} \alpha _4 \rho _n}{1+g_{s-2}\alpha _2 \rho _n }\bigg )\bigg ] \end{aligned}$$

For RC pairing, three different sets of pairs are identified. Let each set receive the corresponding signal with equal probability. The average SSE is attained by considering all three sets of RC pairs. Hence, the SSE is expressed as,

60 $$\begin{aligned} \begin{aligned} S_{HNOMA}^{DL-RC}&= \frac{1}{3}\bigg (\bigg [\frac{1}{2}\log _2(1+g_{s-1} \alpha _1 \rho _n)+\frac{1}{2}\log _2\bigg (1+\frac{g_{s-2} \alpha _2 \rho _n}{1+g_{s-1}\alpha _1 \rho _n }\bigg )\bigg ] \\ &+\bigg [\frac{1}{2}\log _2(1+g_{s-3} \alpha _3 \rho _n)+\frac{1}{2}\log _2\bigg (1+\frac{g_{s-4} \alpha _4 \rho _n}{1+g_{s-3}\alpha _3 \rho _n }\bigg ) \bigg ] \\ &+\bigg [\frac{1}{2}\log _2(1+g_{s-1} \alpha _1 \rho _n)+\frac{1}{2}\log _2\bigg (1+\frac{g_{s-3} \alpha _3 \rho _n}{1+g_{s-1}\alpha _1 \rho _n }\bigg ) \bigg ]\\ &+\bigg [\frac{1}{2}\log _2(1+g_{s-2} \alpha _2 \rho _n)+\frac{1}{2}\log _2\bigg (1+\frac{g_{s-4} \alpha _4 \rho _n}{1+g_{s-2}\alpha _2 \rho _n }\bigg ) \bigg ] \\ &+ \bigg [\frac{1}{2}\log _2(1+g_{s-1} \alpha _1 \rho _n)+\log _2\bigg (1+\frac{g_{s-4} \alpha _4 \rho _n}{1+g_{s-1}\alpha _1 \rho _n }\bigg ) \bigg ]\\ &+\bigg [\frac{1}{2}\log _2(1+g_{s-2} \alpha _2 \rho _n)+\frac{1}{2}\log _2\bigg (1+\frac{g_{s-3} \alpha _3 \rho _n}{1+g_{s-2}\alpha _2 \rho _n }\bigg ) \bigg ]\bigg ) \end{aligned} \end{aligned}$$

where the fraction 1/3 corresponds to the number of sets considered in the RC pairing. The DL-NOMA for $M=4$, the general formula of SE, without pairing and OMA schemes, is obtained by updating (6), and is expressed as,

61 $$\begin{aligned} R_{s-j}^{DL-NOMA}={\left\{ \begin{array}{ll} \log _2(1+g_{s-1} \alpha _1 \rho _n), & \text {for }j=1.\\ \log _2\bigg (1+\frac{g_{s-j} \alpha _j \rho _n}{1+\sum _{l=1}^{j-1}g_{s-l}\alpha _l \rho _n }\bigg ), & \text {for }j=2,3,4. \end{array}\right. } \end{aligned}$$

The SE expression for conventional DL-NOMA system in a multi-user scenario $(M=4)$ is represented in (61). Specifically, it is obtained by substituting the optimal PA factors derived in the preceding steps into the SE expression of conventional NOMA under the given system model. In this setup, the user closest to the gNB ($\text {UD}_1$) performs SIC to decode and subtract the signals of all other users. For the subsequent user ($\text {UD}_2$), the signal intended for $\text {UD}_1$ is treated as interference, and this procedure continues for the remaining users, where each user considers the signals of users with stronger channel conditions (closer to the gNB) as interference. The corresponding SSE for the general DL-NOMA for $M=4$ system is expressed as,

62 $$\begin{aligned} S_{NOMA}^{DL} = \sum _{j=1}^4 R_{s-j}^{DL-NOMA} \end{aligned}$$

#### Remark

Equation (62) is derived from the SSE expression of the NOMA system with optimal PA substituted. This highlights that optimal PA improves SSE by balancing the power between strong and weak users such that neither user’s rate falls below the minimum QoS requirement, while still maximizing the total throughput.

For DL-HNOMA with HI and *i*SIC, the same formula is used to calculate the SSE for every pairing scheme, with changes in the SE. The changes include the terms corresponding to HI and *i*SIC as elaborated in Sect. 3.2. The SSE for the respective pairing schemes will be obtained by updating (57), (58), (59), and (60).

### Probability of outage of DL-HNOMA

For DL-HNOMA, user pairs are formed, consisting of a user with a strong channel condition and another one with a poor channel condition. The probability of outage for a two-user DL-NOMA system, as derived in Section 4.1, corresponds to this scenario. The only difference is in the SE equation for the individual users, where the SE is reduced by a factor of 1/2 due to the TDMA scheme. Hence, for $\text {UD}_j$ in each pair, the probability of outage is calculated using (23). This involves substituting the expression for $\beta _j$ with the modified formula $\beta _j = 2^{2\tilde{\beta _j}} - 1$, due to the changes in the SE formula in the DL-HNOMA system. For DL-HNOMA with HI and *i*SIC, the methodology used in Section 4.2 is used to address the HI and *i*SIC in the SE of $\text {UD}_j$. The TDMA scheme in DL-HNOMA divides the time resource into two equal sessions. Hence, the individual user’s SE will be reduced by a factor of 1/2. Once the SE is updated, the probability of outage is calculated using (27), with $\beta _j = 2^{2\tilde{\beta _j}} - 1$.

### SSE of UL-HNOMA

The SSE analysis for the UL-HNOMA is presented in this section. For SP pairing, $\text {UD}_2$ and $\text {UD}_4$ perform SIC to decode the data, while $\text {UD}_1$ and $\text {UD}_3$ decode the data directly. Hence, the SSE for SP pairing is expressed as,

63 $$\begin{aligned} S_{HNOMA}^{UL-SP}= & \bigg [\frac{1}{2}\log _2(1+g_{s-2} \alpha _2 \rho _n)+\frac{1}{2}\log _2\bigg (1+\frac{g_{s-1} \alpha _1 \rho _n}{1+g_{s-2}\alpha _2 \rho _n }\ \bigg )\bigg ]\nonumber \\ & +\bigg [\frac{1}{2}\log _2(1+g_{s-4} \alpha _4 \rho _n)+\frac{1}{2}\log _2\bigg (1+\frac{g_{s-3} \alpha _3 \rho _n}{1+g_{s-4}\alpha _4 \rho _n }\bigg ) \bigg ] \end{aligned}$$

For SW pairing, $\text {UD}_4$ and $\text {UD}_3$ perform SIC to decode the data, while $\text {UD}_1$ and $\text {UD}_2$ decode the data directly. Hence, the SSE for SW pairing is expressed as,

64 $$\begin{aligned} S_{HNOMA}^{UL-SW}= & \bigg [\frac{1}{2}\log _2(1+g_{s-4} \alpha _4 \rho _n)+\frac{1}{2}\log _2\bigg (1+\frac{g_{s-1} \alpha _1 \rho _n}{1+g_{s-4}\alpha _4 \rho _n }\bigg )\bigg ]\nonumber \\ & +\bigg [\frac{1}{2}\log _2(1+g_{s-3} \alpha _3 \rho _n)+\frac{1}{2}\log _2\bigg (1+\frac{g_{s-2} \alpha _2 \rho _n}{1+g_{s-3}\alpha _3 \rho _n }\bigg )\bigg ] \end{aligned}$$

For IB pairing, $\text {UD}_3$ and $\text {UD}_4$ perform SIC to decode the data, while $\text {UD}_1$ and $\text {UD}_2$ decode the data directly. Hence, the SSE for IB pairing is expressed as,

65 $$\begin{aligned} S_{HNOMA}^{UL-IB}= & \bigg [\frac{1}{2}\log _2(1+g_{s-3} \alpha _3 \rho _n)+\frac{1}{2}\log _2\bigg (1+\frac{g_{s-1} \alpha _1 \rho _n}{1+g_{s-3}\alpha _3 \rho _n } \bigg )\bigg ]\nonumber \\ & +\bigg [\frac{1}{2}\log _2(1+g_{s-4} \alpha _4 \rho _n)+\frac{1}{2}\log _2\bigg (1+\frac{g_{s-2} \alpha _2 \rho _n}{1+g_{s-4}\alpha _4 \rho _n }\bigg )\bigg ] \end{aligned}$$

The average SSE is attained by considering all three sets of RC pairs. Hence, for RC pairing, the SSE is expressed as,

66 $$\begin{aligned} \begin{aligned} S_{HNOMA}^{UL-RC}&= \frac{1}{3}\bigg (\bigg [\frac{1}{2}\log _2(1+g_{s-2} \alpha _2 \rho _n)+\frac{1}{2}\log _2\bigg (1+\frac{g_{s-1} \alpha _1 \rho _n}{1+g_{s-2}\alpha _2 \rho _n }\bigg )\bigg ] \\ &+\bigg [\frac{1}{2}\log _2(1+g_{s-4} \alpha _4 \rho _n)+\frac{1}{2}\log _2\bigg (1+\frac{g_{s-3} \alpha _3 \rho _n}{1+g_{s-4}\alpha _4 \rho _n }\bigg ) \bigg ] \\ &+\bigg [\frac{1}{2}\log _2(1+g_{s-3} \alpha _3 \rho _n)+\frac{1}{2}\log _2\bigg (1+\frac{g_{s-1} \alpha _1 \rho _n}{1+g_{s-3}\alpha _3 \rho _n }\bigg ) \bigg ]\\ &+\bigg [\frac{1}{2}\log _2(1+g_{s-4} \alpha _4 \rho _n)+\frac{1}{2}\log _2\bigg (1+\frac{g_{s-2} \alpha _2 \rho _n}{1+g_{s-4}\alpha _4 \rho _n }\bigg ) \bigg ] \\ &+ \bigg [\frac{1}{2}\log _2(1+g_{s-4} \alpha _4 \rho _n)+\log _2\bigg (1+\frac{g_{s-1} \alpha _1 \rho _n}{1+g_{s-4}\alpha _4 \rho _n }\bigg ) \bigg ]\\ &+\bigg [\frac{1}{2}\log _2(1+g_{s-3} \alpha _3 \rho _n)+\frac{1}{2}\log _2\bigg (1+\frac{g_{s-2} \alpha _2\rho _n}{1+g_{s-3}\alpha _3 \rho _n }\bigg ) \bigg ]\bigg ) \end{aligned} \end{aligned}$$

where the fraction 1/3 corresponds to the number of sets considered in the RC pairing. The UL-NOMA for $M=4$, the general formula of SE, without pairing and OMA schemes is obtained by updating (13), and is expressed as,

67 $$\begin{aligned} R_{s-j}^{UL-NOMA}={\left\{ \begin{array}{ll} \log _2(1+g_{s-4} \alpha _4 \rho _n), & \text {for }j=4.\\ \log _2\bigg (1+\frac{g_{s-j} \alpha _j \rho _n}{1+\sum _{l=j+1}^{4}g_{s-l}\alpha _l \rho _n }\bigg ), & \text {for }j=1,2,3. \end{array}\right. } \end{aligned}$$

The corresponding SSE for the general UL-NOMA for $M=4$ system is expressed as,

68 $$\begin{aligned} S_{NOMA}^{UL} = \sum _{j=1}^4 R_{s-j}^{UL-NOMA} \end{aligned}$$

Similar to DL-HNOMA, the SE of $\text {UD}_j$ in UL-HNOMA is reduced by a factor of 1/2 due to the TDMA scheme. For UL-HNOMA with HI and *i*SIC, to calculate SSE, a similar analytical approach, as outlined in Section 5.4, is adapted to effectively address the HI and *i*SIC. By utilizing the updated SE, it is possible to compute the SSE by employing (63), (64), (65), and (66) for each pairing scheme.

### Probability of outage of UL-HNOMA

In UL-HNOMA, pairs of users are created, consisting of one user with good channel conditions and another with poor channel conditions. The outage probability for a two-user UL-NOMA system, as discussed in Section 4.3, pertains to this situation. The only variation lies in the SE for each user, where the SE is reduced by a factor of 1/2 due to the TDMA approach. Hence, for $\text {UD}_j$ with strong channel conditions in each pair, the probability of outage is calculated using (38) and for $\text {UD}_j$ with poor channel conditions in each pair, the probability of outage is calculated using (47). This involves substituting the expression for $\beta _j$ with the modified formula $\beta _j = 2^{2\tilde{\beta _j}} - 1$, due to the changes in the SE formula in the UL-HNOMA system. For UL-HNOMA with HI and *i*SIC, the methodology used in Section 4.4 is used to address the HI and *i*SIC in the SE of $\text {UD}_j$. The TDMA scheme in DL-HNOMA divides the time resource into two equal sessions. Hence, the individual user’s SE will be reduced by a factor of 1/2. Once the SE is updated, the probability of outage is calculated using (53) for the user with a strong channel and using (56) for the user with a poor channel. This also involves substituting the expression for $\beta _j$ with the modified formula $\beta _j = 2^{2\tilde{\beta _j}} - 1$.

## SSE enhancement through optimized PA factors

The optimization of PA factors requires finding a balance between enhancing SSE and ensuring user fairness. Focusing solely on maximizing SSE will results in unfair power distribution. The objective of the work is to maximize the SSE in the system with guaranteed fairness for every user. Hence, outage conditions are considered to derive the optimal range for the PA factors, which provides maximum SSE. The SE minimum meets the fairness demands of individual users. This section formulates the optimal and suboptimal range of PA factors to enhance the SSE while ensuring minimum fairness for DL and UL-NOMA systems. Hence, the objective function for DL and UL-NOMA can be formulated as,

69a $$\begin{aligned} & \max _{\alpha _j} \; S_{\text{ NOMA }}^{\text{ DL/UL }}, \end{aligned}$$

69b $$\begin{aligned} & Subject\;to: \;\;\Sigma _{j=1}^{2}\alpha _j=1, \end{aligned}$$

69c $$\begin{aligned} & \;\; 0<\alpha _1<\alpha _2<1, \end{aligned}$$

69d $$\begin{aligned} & \;\;R^{DL/UL}_{s-1}>\tilde{\beta }_1, R^{DL/UL}_{s-2}>\tilde{\beta }_{2}, R_{UD_1}^{UD_2}>\tilde{\beta }_{2}, \end{aligned}$$

In the case of DL-NOMA, the SSE is reformulated by substituting (6) into (7). After rearranging, it is expressed as,

70 $$\begin{aligned} S^{NOMA}_{DL} = \log _2\bigg (\frac{(1+g_{s-2}\rho _n)(1+g_{s-1} \alpha _1 \rho _n)}{(1+g_{s-2}\alpha _1 \rho _n)}\bigg ) \end{aligned}$$

Since $g_{s-1}>g_{s-2}$, it can be observed that the SSE is monotonically increasing with respect to $\alpha _1$. Thus, the optimal range of PA factors that maximizes the SSE, ensuring minimum fairness, is the same as the range for $\alpha _1$, which is obtained by rearranging the outage conditions for DL-NOMA and replacing $\alpha _2$ with $(1-\alpha _1)$. It is expressed as,

71 $$\begin{aligned} \underbrace{\frac{\beta _1}{g_{s-1}\rho _n}}_{\alpha ^{DL-min}_{1}} \le \alpha _1\le \underbrace{\frac{g_{s-1}\rho _n-\beta _{2}}{g_{s-1}\rho _n(\beta _{2}+1)}}_{\alpha ^{DL-max}_{1}} \end{aligned}$$

In the optimal range of $\alpha _1$, the highest value provides maximum SSE. Hence, the optimal value for $\alpha _1$ is expressed as,

72 $$\begin{aligned} \alpha ^{DL-opt}_{1}=\frac{g_{s-1}\rho _n-\beta _{2}}{g_{s-1}\rho _n(\beta _{2}+1)} \end{aligned}$$

The suboptimal value for $\alpha _1$ in DL-NOMA is obtained by calculating the average of its range given in (71), and it is expressed as^39^,

73 $$\begin{aligned} \alpha ^{DL-subopt}_{1}= \frac{\alpha ^{DL-max}_{1}+\alpha ^{DL-min}_{1}}{2} \end{aligned}$$

Similarly, the optimal range for $\alpha _1$ in the case of UL-NOMA is expressed as,

74 $$\begin{aligned} \underbrace{\frac{\beta _1(1+ g_{s-2}\rho _n)}{(g_{s-1}+g_{s-2}\beta _1)\rho _n}}_{\alpha ^{UL-min}_{1}} \le \alpha _1\le \underbrace{\frac{g_{s-2}\rho _n-\beta _{2}}{g_{s-2}\rho _n}}_{\alpha ^{UL-max}_{1}} \end{aligned}$$

The optimal value for $\alpha _1$ in the case of UL-NOMA is the maximum value in (74) and it is expressed as,

75 $$\begin{aligned} \alpha ^{UL-opt}_{1}={\frac{g_{s-2}\rho _n-\beta _{2}}{g_{s-2}\rho _n}} \end{aligned}$$

The suboptimal value for $\alpha _1$ in UL-NOMA is obtained by calculating the average of its range given in (74), and it is expressed as^39^,

76 $$\begin{aligned} \alpha ^{UL-subopt}_{1}= \frac{\alpha ^{UL-max}_{1}+\alpha ^{UL-min}_{1}}{2} \end{aligned}$$

In both DL and UL-NOMA, the optimal and suboptimal values of $\alpha _2$ is determined by subtracting the optimal and suboptimal values of $\alpha _1$ from unity. Through similar analysis, optimal and suboptimal PA factors can be derived for DL and UL-NOMA with HI and *i*SIC.

## Results and discussion

This section presents the analysis of the probability of outage and SSE for DL, UL-NOMA, and HNOMA systems under various conditions. Different values of *m* are considered to address the impact of different channel scenarios. The probability of outage analysis also explains the role of HI and *i*SIC. The range of values considered for the HI parameter is 0.08 to 0.1 and it is denoted as $\lambda$.^35^. The value of the *i*SIC parameter $\varepsilon$ considered is 0.1^37^. The performance analysis of different pairing strategies is demonstrated using plots of the average probability of outage and SSE. The SSE for DL and UL-HNOMA systems using optimized PA factors is plotted corresponding to different pairing schemes. To perform the Monte Carlo simulation, a large set of random channel coefficients is considered. The simulation software used is MATLAB R2023b. In all the graphs, simulation results are illustrated with symbols, while theoretical results are illustrated with solid lines. The values of different parameters considered for DL and UL-NOMA system simulation are listed in Table 3. In the proposed work, the values such as *m*, $\alpha _j$, $P_t$, $\varepsilon$, and $\lambda$ are selected based on established ranges found in the existing literature on NOMA systems and practical scenarios for 5G and 6G networks. For example, *m* values between 1 and 3 are typical for modeling urban microcell environments, representing conditions from Rayleigh to Rician fading. HI factors and *i*SIC parameters were chosen to reflect typical impairment levels observed in real-time experiments and analyses. According to 3GPP LTE specifications, the total error vector magnitude (EVM) for transmitters is required to be in the range $\lambda \in [0.08, 0.175]$, with smaller values needed to support higher SE^40^. The transmit power range values we used, ranging from -20 dBm to 50 dBm, are representative of typical link conditions in cellular and Internet of Things (IoT) applications.

**Table 3:** System parameters for simulation of DL and UL-NOMA.

Parameter	Values
Transmit power for DL and UL $(P_{t})$	$-20$ to 50 dBm
Distance of $\text {UD}_1$ from gNB ($\delta _{S-1}$)	300 meters
Distance of $\text {UD}_2$ from gNB ($\delta _{S-2}$)	600 meters
Path loss factor ($\eta$)	4
Number of random channel coefficients (*N*)	$10^7$
*i*SIC parameter ($\varepsilon$)	0 and 0.1
HI parameter ($\lambda$)	0.08 and 0.1
Minimum demanded SE for $\text {UD}_1$ ($\tilde{\beta }_1$), and $\text {UD}_2$ ($\tilde{\beta }_2$)	1 bps/Hz

**Fig. 7:**
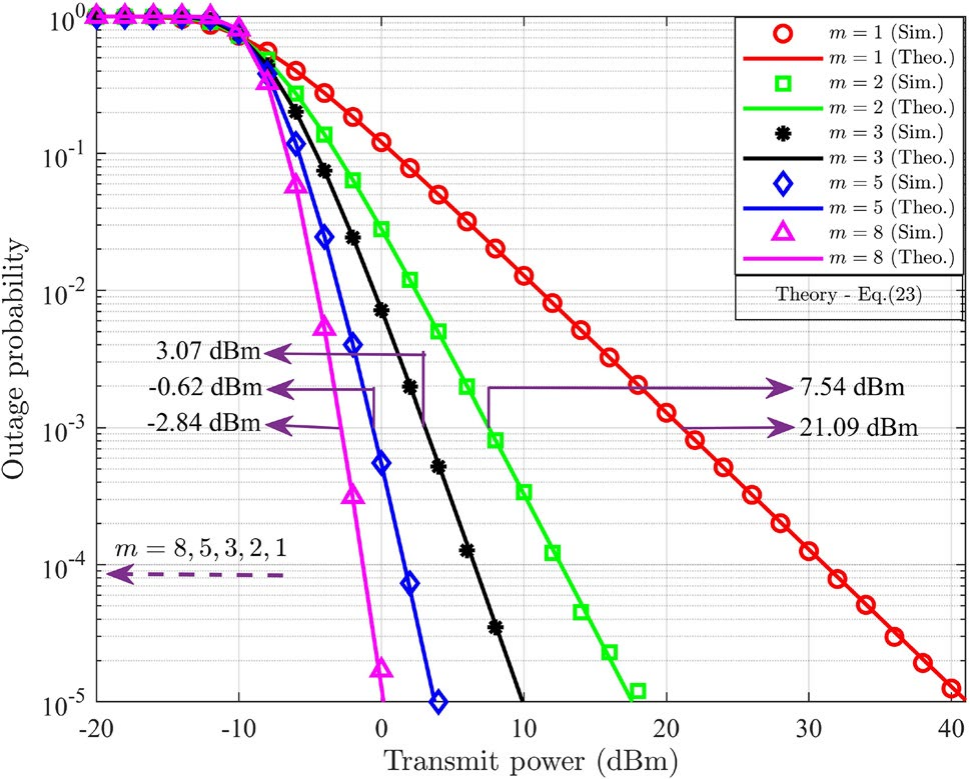
Probability of outage comparison for $\text {UD}_1$ of DL-NOMA.

**Fig. 8:**
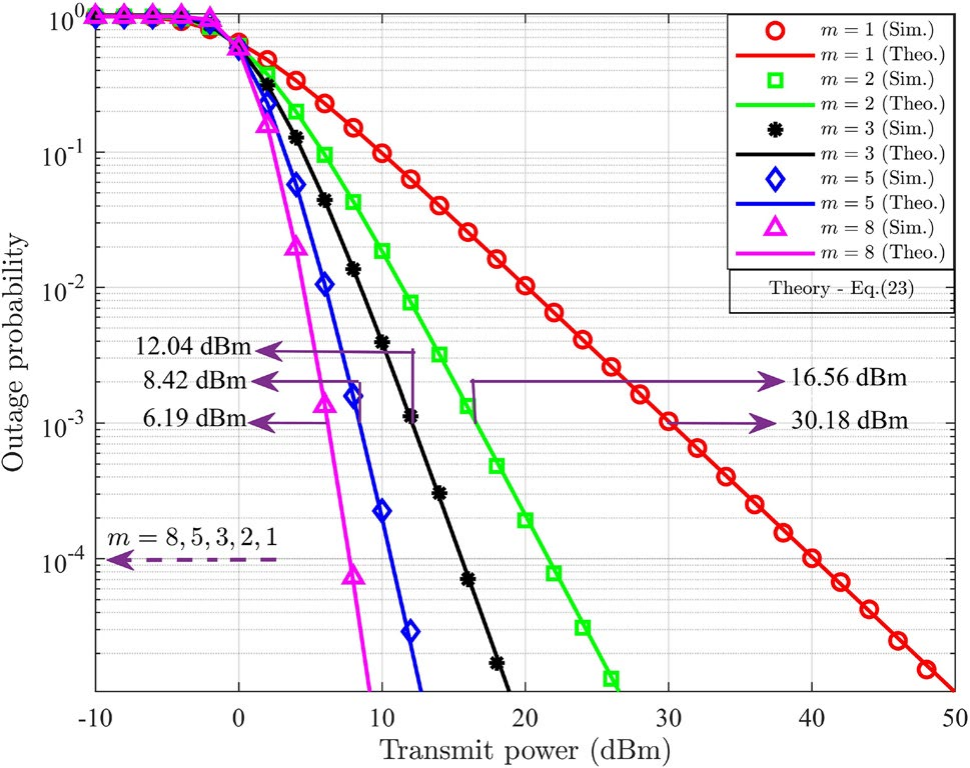
Probability of outage comparison for $\text {UD}_2$ of DL-NOMA.

The probability of outage for $\text {UD}_1$ in the two-user DL-NOMA system is depicted in Fig. 7. The graph is plotted for different values of *m*. It has been observed that an increase in the value of *m* correlates with a reduction in outage occurrences, due to the dominance of the LOS component in the multipath communication. For both $\text {UD}_1$ and $\text {UD}_2$, the theoretical results are plotted using (23). For comparison purposes, a target outage of $10^{-3}$ is considered for both users. The minimum $P_t$ required to achieve the target outage is $\approx$21.09 dBm, $\approx$7.54 dBm, $\approx$3.07 dBm, $\approx$-0.62 dBm, and $\approx$-2.84 dBm for *m* equal to 1, 2, 3, 5, and 8, respectively. Hence, the scenario, *m* equal to 8, requires $\approx 2.22$ dBm, $\approx 5.91$ dBm, $\approx 10.38$ dBm, and $\approx 23.93$ dBm, less $P_t$ than *m* equal to 5, 3, 2, and 1 scenarios, respectively. The probability of outage for $\text {UD}_2$ in the two-user DL-NOMA system is depicted in Fig. 8. As the value of *m* increases, the outage for $\text {UD}_2$ decreases. The minimum required $P_t$ to achieve the target outage is $\approx$30.18 dBm, $\approx$16.56 dBm, $\approx$12.04 dBm, $\approx$8.42 dBm, and $\approx$6.19 dBm for the condition *m* equal to 1, 2, 3, 5, and 8. Hence, the scenario, *m* equal to 8, requires $\approx 2.23$ dBm, $\approx 5.85$ dBm, $\approx 10.37$ dBm, and $\approx 23.99$ dBm, less $P_t$ than *m* equal to 5, 3, 2, and 1 scenarios, respectively. In comparison to $\text {UD}_2$, $\text {UD}_1$ exhibits better performance in terms of outage. For *m* equal to 8, $\text {UD}_1$ requires $\approx 9.03$ dBm less $P_t$ than $\text {UD}_2$. This is due to the favorable channel conditions for $\text {UD}_1$ compared to $\text {UD}_2$ over the given distances. The $P_t$ required by DL-NOMA users $\text {UD}_1$ and $\text {UD}_2$ to achieve an outage probability of $10^{-3}$ for varying values of *m*, $\lambda$, and $\varepsilon$ is illustrated in Table 4.

**Fig. 9:**
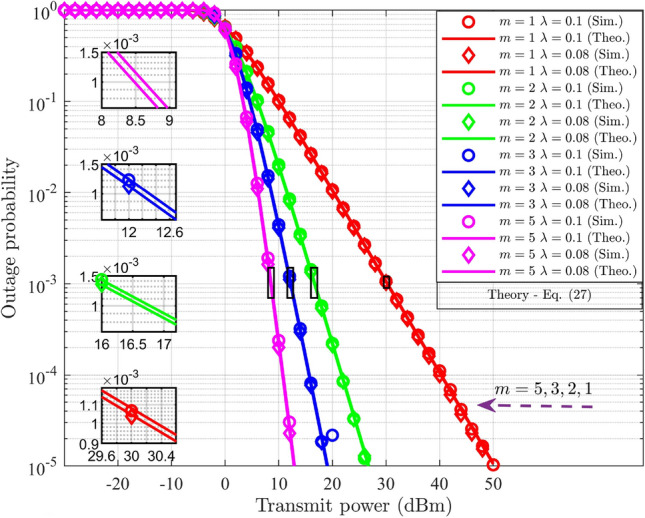
Probability of outage comparison for $\text {UD}_1$ with HI and *i*SIC of DL-NOMA.

The probability of outage for $\text {UD}_1$ under HI and *i*SIC in two-user DL-NOMA is illustrated in Fig. 9. The graph is plotted for different values of *m*, equal to 1, 2, 3, and 5. For both $\text {UD}_1$ and $\text {UD}_2$, the theoretical results are plotted using (27). To analyze the impact of *i*SIC, the value of $\varepsilon$ is considered to be 0.1. The value of $\lambda$ varies from 0.08 to 0.1, and the corresponding outage is plotted. From Table 4, it is observed that, for each *m*, as it $\lambda$ increases, the outage for $\text {UD}_1$ increases. The condition *m* equal to 5, with $\lambda$ equal to 0.08, provides the minimum outage compared to other scenarios. The scenario, *m* equal to 5, with $\lambda$ equal to 0.08, requires $\approx 3.68$ dBm, $\approx 8.14$ dBm, and $\approx 21.68$ dBm, less $P_t$ than *m* equal to 3, 2, and 1, with $\lambda$ equal to 0.08, scenarios, respectively. For *m* equal to 5, when $\lambda$ increases to 0.1, $\text {UD}_1$ requires an additional $P_t$ of $\approx 0.45$ dBm to achieve the target outage.

**Fig. 10:**
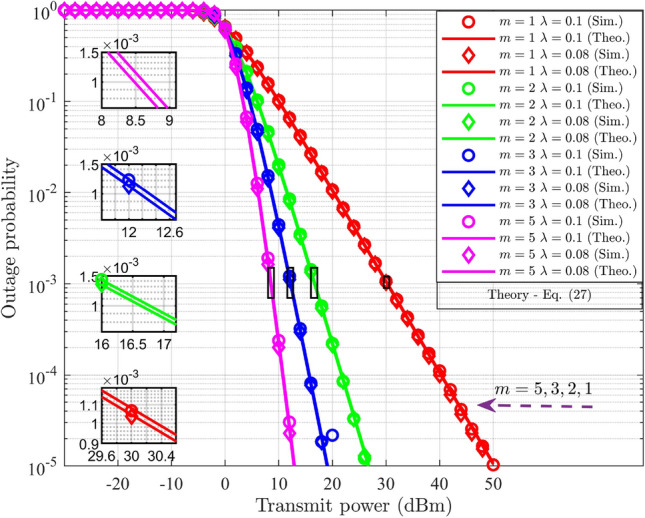
Probability of outage comparison for $\text {UD}_2$ with HI of DL-NOMA.

Figure 10 illustrates the probability of outage for $\text {UD}_2$ under HI, for different values of *m*. Since $\text {UD}_2$ does not perform SIC, $\varepsilon$ is considered to be zero. From Table 4, it can be observed that, for $\text {UD}_2$, as the value of *m* increases, the outage decreases, while the outage increases as the value of $\lambda$ increases. For the scenario, *m* equal to 5, with $\lambda$ equal to 0.08, requires $\approx 3.68$ dBm, $\approx 8.14$ dBm, and $\approx 21.71$ dBm, less $P_t$ than *m* equal to 3, 2, and 1, with $\lambda$ equal to 0.08, scenarios, respectively. For *m* equal to 5, when $\lambda$ increases to 0.1, $\text {UD}_2$ requires an additional $P_t$ of $\approx 0.13$ dBm to achieve the target outage. Here, only the impact of $\lambda$ is considered; hence, the difference in outage is minimal for a particular value of *m* when $\lambda$ changes. In comparison to $\text {UD}_2$, $\text {UD}_1$ shows better performance in terms of outage. For *m* equal to 5, with $\lambda$ equal to 0.08 $\text {UD}_1$ requires $\approx 7.39$ dBm less $P_t$ than $\text {UD}_2$.

**Table 4:** $P_t$ required by DL-NOMA users $\text {UD}_1$ and $\text {UD}_2$ to achieve an outage probability of $10^{-3}$ for varying values of *m*, $\lambda$, and $\varepsilon$.

*m*	$\lambda$	Required $P_t$ (dBm) at $\text {UD}_1$ ($\varepsilon = 0$)	Required $P_t$ (dBm) at $\text {UD}_1$ ($\varepsilon = 0.1$)	Required $P_t$ (dBm) at $\text {UD}_2$ ($\varepsilon = 0$)
1	0	21.09	–	30.18
	0.08	–	22.77	30.19
	0.1	–	23.23	30.33
2	0	7.54	–	16.56
	0.08	–	9.23	16.62
	0.1	–	9.68	16.77
3	0	3.07	–	12.04
	0.08	–	4.77	12.16
	0.1	–	5.21	12.30
5	0	−0.62	–	8.42
	0.08	–	1.09	8.48
	0.1	–	1.54	8.61
8	0	−2.84	–	6.19

**Fig. 11:**
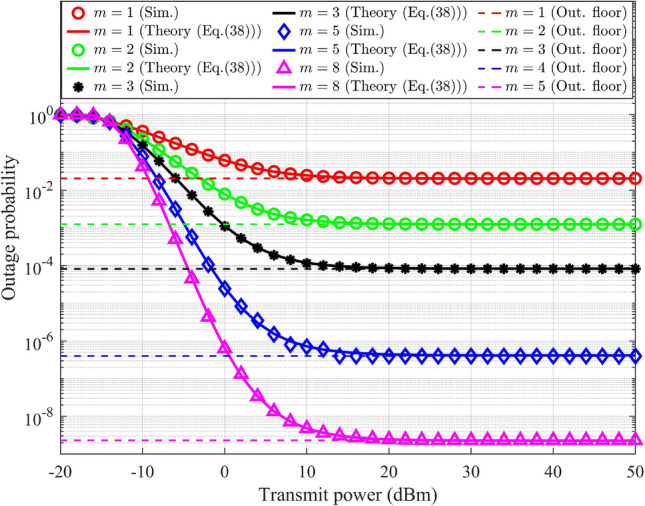
Probability of outage comparison for $\text {UD}_1$ of UL-NOMA.

The probability of outage for $\text {UD}_1$ for different values of *m* in the two-user UL-NOMA is illustrated in Fig. 11. In the UL-NOMA system, after a particular level of $P_t$, the outage reaches a saturation level, called the outage floor. It is observed that, as the value of *m* increases, the outages and outage floor values decrease. For almost every value of *m*, $\text {UD}_1$ reaches the outage floor at a $P_t$ of $\approx$18 dBm. The outage floors are $2\times 10^{-2}, 1.23\times 10^{-3}, 8.11\times 10^{-5}, 4\times 10^{-7},$ and $2.32\times 10^{-9}$ for the respective *m* values of 1,2,3,5 and 8. The theoretical results are plotted using (38).

**Fig. 12:**
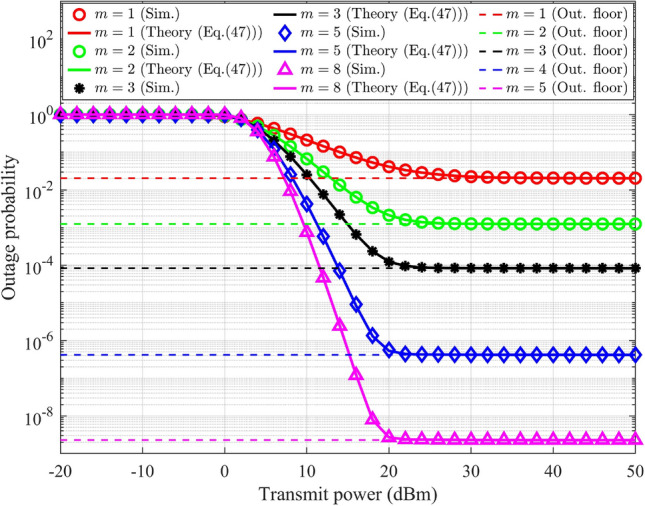
Probability of outage comparison for $\text {UD}_2$ of UL-NOMA.

The probability of outage for $\text {UD}_2$ for different values of *m* in the two-user UL-NOMA is depicted in Fig. 12. The theoretical results are plotted using (47). Similar to $\text {UD}_1$, as the value of *m* increases, the outages and outage floor values of $\text {UD}_2$ decrease. For almost every value of *m*, $\text {UD}_2$ reaches the outage floor at a $P_t$ of $\approx$22 dBm. The outage floors are $2.06\times 10^{-2}, 1.23\times 10^{-3}, 8.28\times 10^{-5}, 4.17\times 10^{-7},$ and $2.29\times 10^{-9}$ for the respective *m* values of 1,2,3,5 and 8. The outage floor values for $\text {UD}_1$ and $\text {UD}_2$ are nearly equal, but $\text {UD}_2$ requires an additional $P_t$ of $\approx$ 4 dBm compared to $\text {UD}_1$ to reach the outage floor. The outage floor comparison of UL-NOMA users $\text {UD}_1$ and $\text {UD}_2$ for a fixed $P_t$ (dBm) under varying values of *m*, $\lambda$, and $\varepsilon$ is detailed in Table 5.

**Fig. 13:**
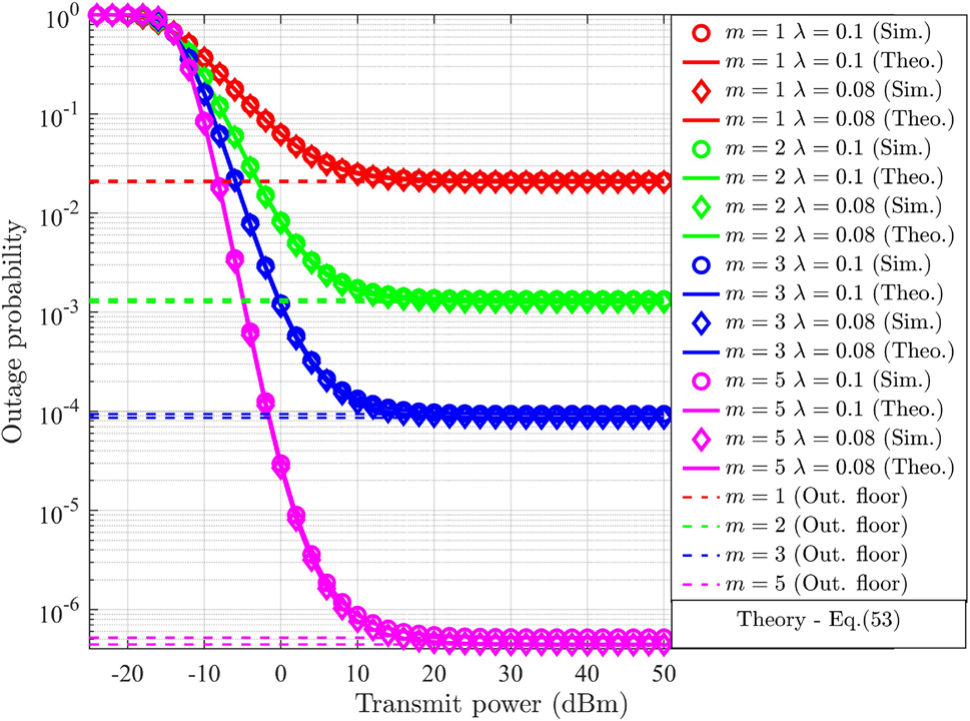
Probability of outage comparison for $\text {UD}_1$ with HI of UL-NOMA.

**Fig. 14:**
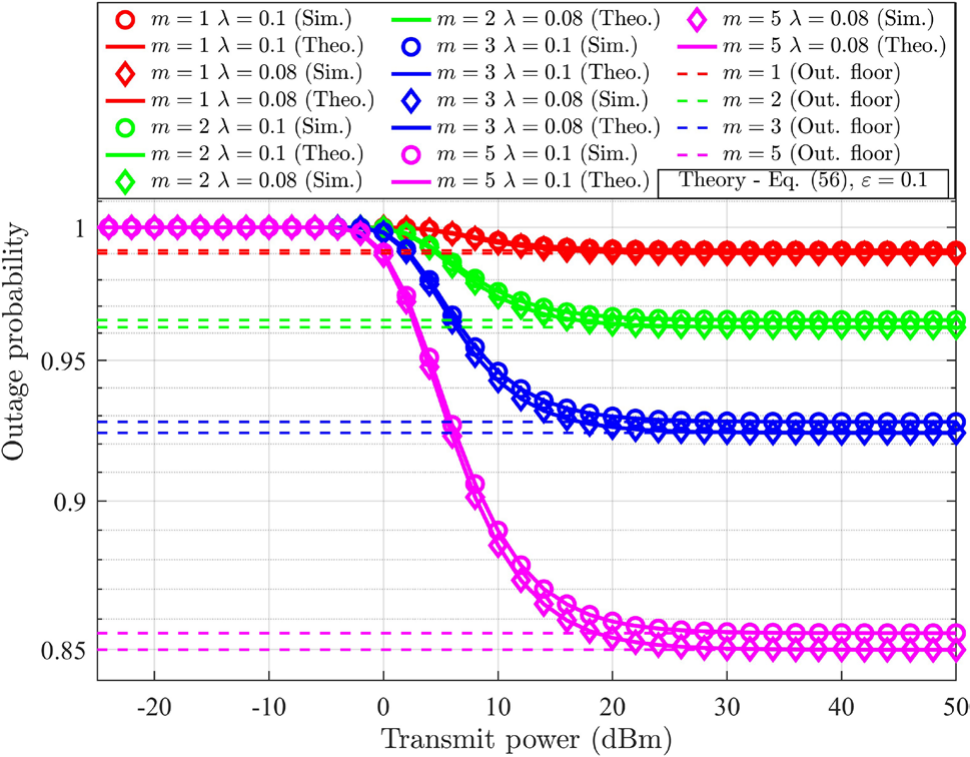
Probability of outage comparison for $\text {UD}_2$ with HI and *i*SIC of UL-NOMA.

Figure 13 depicts the probability of outage of $\text {UD}_1$ in the UL-NOMA system under HI. The theoretical results are plotted using (53). In the UL-NOMA system, the gNB directly decodes $\text {UD}_1$’s data; hence, in this case $\varepsilon$ is considered zero. For each value of *m*, $\lambda$ is varied from 0.08 to 0.1, and the corresponding outage is plotted. It is observed that an increase in the value of *m* leads to a reduction in outage, whereas an increase in the value of $\lambda$ corresponds with a rise in outage. Compared to other scenarios, $m=5, \lambda =0.08$ provides the minimum outage for $\text {UD}_1$. For *m* equal to 5, when $\lambda$ changes from 0.08 to 0.1 the outage floor increases from $\approx 4.43 \times 10^{-7}$ to $\approx 5.17 \times 10^{-7}$. Figure 14 depicts $\text {UD}_2$’s probability of outage in the UL-NOMA under HI and *i*SIC. Here, the theoretical results are plotted using (56). For each case of *m*, $\lambda$ is varied from 0.08 to 0.1, and $\varepsilon$ is fixed to 0.1. Compared to other scenarios, $m=5, \lambda =0.08$ provides the minimum outage for $\text {UD}_2$ . For *m* equal to 5, when $\lambda$ changes from 0.08 to 0.1 the outage floor increases from $\approx 8.49 \times 10^{-1}$ to $\approx 8.55 \times 10^{-1}$. Table 5 captures the observations corresponding to Figs. 13 and 14. For the minimum outage condition with HI, the outage floor for $\text {UD}_1$ is $\approx 4.43 \times 10^{-7}$ and for $\text {UD}_2$ is $\approx 8.49 \times 10^{-1}$ and the difference is due to the *i*SIC process.

**Table 5:** Outage floor comparison of UL-NOMA users $\text {UD}_1$ and $\text {UD}_2$ for a fixed $P_t$ (dBm) under varying values of *m*, $\lambda$, and $\varepsilon$.

*m*	$\lambda$	Outage floor at $\text {UD}_1$ for $P_t=18$ dBm ($\varepsilon = 0$)	Outage floor at $\text {UD}_2$ for $P_t=22$ dBm ($\varepsilon = 0$)	Outage floor at $\text {UD}_2$ for $P_t=22$ dBm ($\varepsilon = 0.1$)
1	0	$2\times 10^{-2}$	$2.06\times 10^{-2}$	–
	0.08	$2.06\times 10^{-2}$	–	$9.9\times 10^{-1}$
	0.1	$2.13\times 10^{-2}$	–	$9.92\times 10^{-1}$
2	0	$1.23\times 10^{-3}$	$1.23\times 10^{-3}$	–
	0.08	$1.26\times 10^{-3}$	–	$9.62\times 10^{-1}$
	0.1	$1.35\times 10^{-3}$	–	$9.64\times 10^{-1}$
3	0	$8.11\times 10^{-5}$	$8.28\times 10^{-5}$	–
	0.08	$8.59\times 10^{-5}$	–	$9.23\times 10^{-1}$
	0.1	$9.37\times 10^{-5}$	–	$9.28\times 10^{-1}$
5	0	$4\times 10^{-7}$	$4.17\times 10^{-7}$	–
	0.08	$4.43\times 10^{-7}$	–	$8.49\times 10^{-1}$
	0.1	$5.17\times 10^{-7}$	–	$8.55\times 10^{-1}$
8	0	$2.32\times 10^{-9}$	$2.29\times 10^{-9}$	–

For the performance analysis of user pairing schemes, the average outage probability is calculated by dividing the users in each pair into two categories. The set of users located closer to the gNB in every pair is denoted as $\text {UD}_s$ and represents the users with strong channel conditions. For SP pairing, $\text {UD}_s$ = {$\text {UD}_1$, $\text {UD}_3$}, for IB pairing, $\text {UD}_s$ = {$\text {UD}_1$, $\text {UD}_2$}, for SW pairing $\text {UD}_s$ = {$\text {UD}_1$, $\text {UD}_2$}, and for RC pairing $\text {UD}_s$ = {$\text {UD}_1$, $\text {UD}_2$, $\text {UD}_3$}. The set of users positioned far from the gNB in each pair is denoted as $\text {UD}_w$ and represents the users with poor channel conditions. For SP pairing, $\text {UD}_w$ = {$\text {UD}_2$, $\text {UD}_4$}, for IB pairing, $\text {UD}_w = \{\text {UD}_3$, $\text {UD}_4$}, for SW pairing $\text {UD}_w$ = {$\text {UD}_3$, $\text {UD}_4$}, and for RC pairing $\text {UD}_w = \{\text {UD}_2$, $\text {UD}_3$, $\text {UD}_4$}. In each scheme, the users are considered as pairs. Since the number of users considered is $M=4$, the number of pairs is 2. Each pair consists of one user with strong channel condition and another one with weak channel condition. The set $\text {UD}_s$ considers the users with strong channel conditions, and the set $\text {UD}_w$ considers users with weak channel conditions. Since each pair is similar to a two-user NOMA system, the probability of each user can be calculated accordingly. The average probability of outage of $\text {UD}_s$ in each scheme is calculated by taking the average of the calculated outage of every user included in the set of $\text {UD}_s$. Similarly, the average probability of outage of $\text {UD}_w$ in each scheme is calculated by taking the average of the calculated outage of every user included in the set of $\text {UD}_w$. Hence, the outage results for users with strong channel conditions in each pair are computed by averaging $\text {UD}_s$’s outages. Conversely, the outage results for users with poor channel conditions in each pair are computed by averaging $\text {UD}_w$’s outages. The values of different parameters considered for DL and UL-HNOMA system simulations are listed in Table 6.

**Fig. 15:**
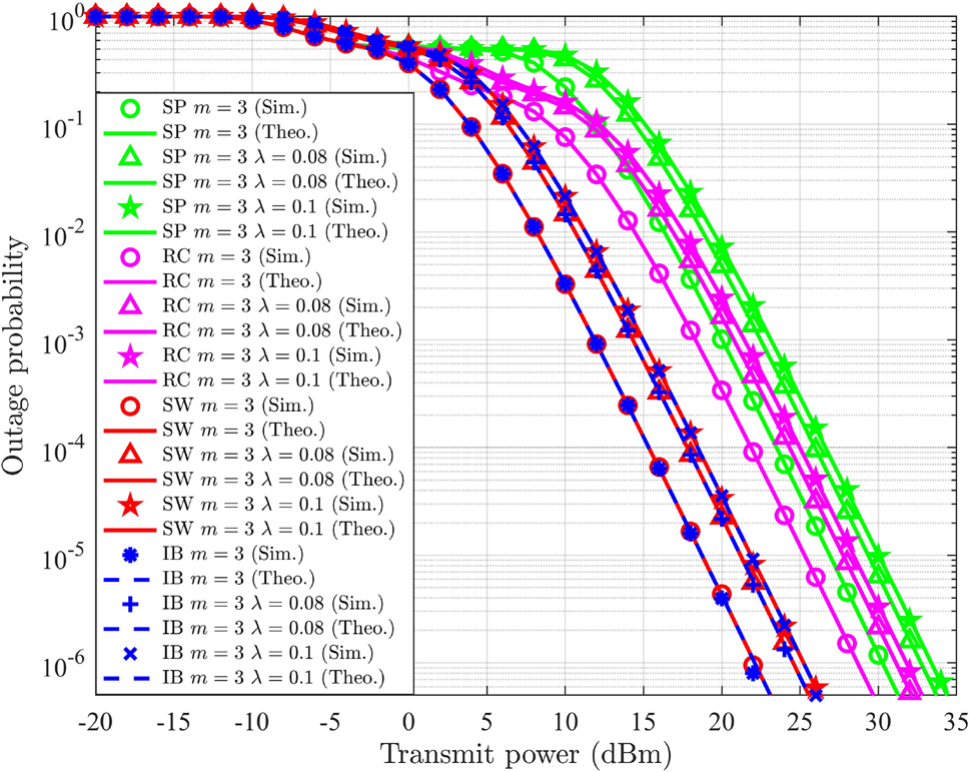
Comparison of average outage probability for $\text {UD}_s$ under various pairing schemes in DL-HNOMA.

**Fig. 16:**
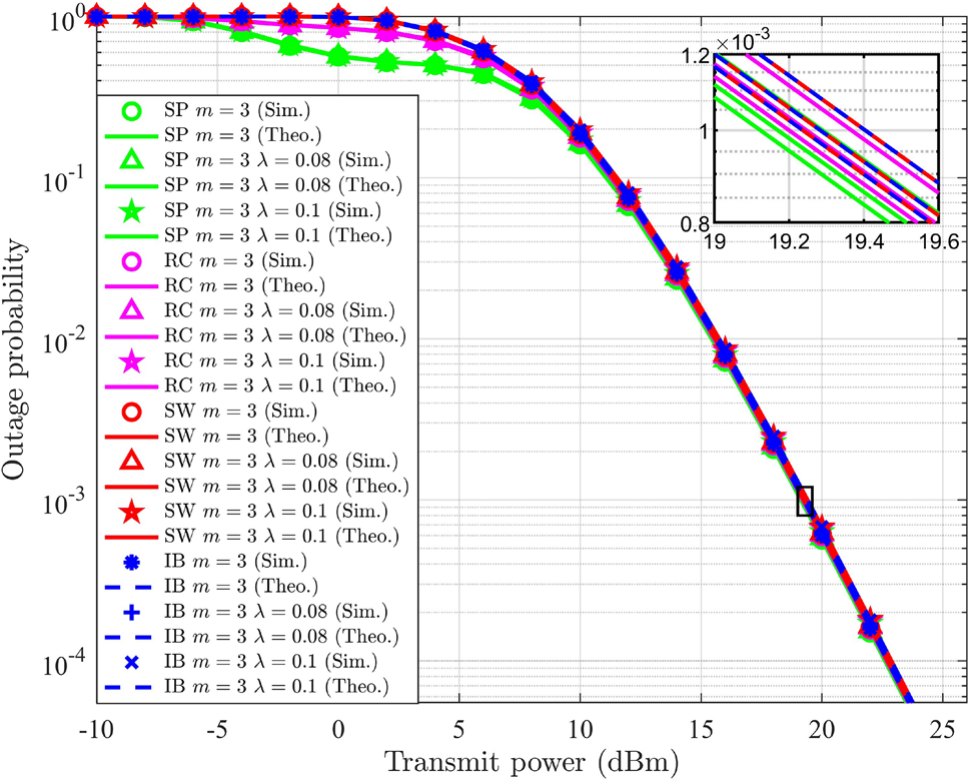
Comparison of average outage probability for $\text {UD}_w$ under various pairing schemes in DL-HNOMA.

**Fig. 17:**
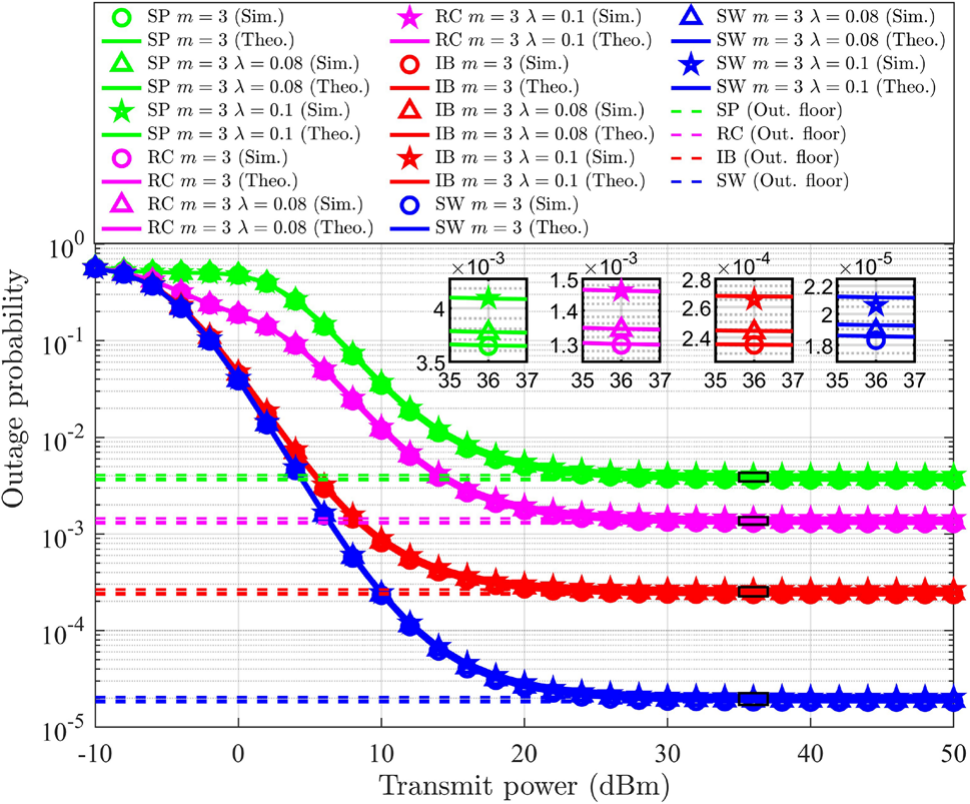
Comparison of average outage probability for $\text {UD}_s$ under various pairing schemes in UL-HNOMA.

**Fig. 18:**
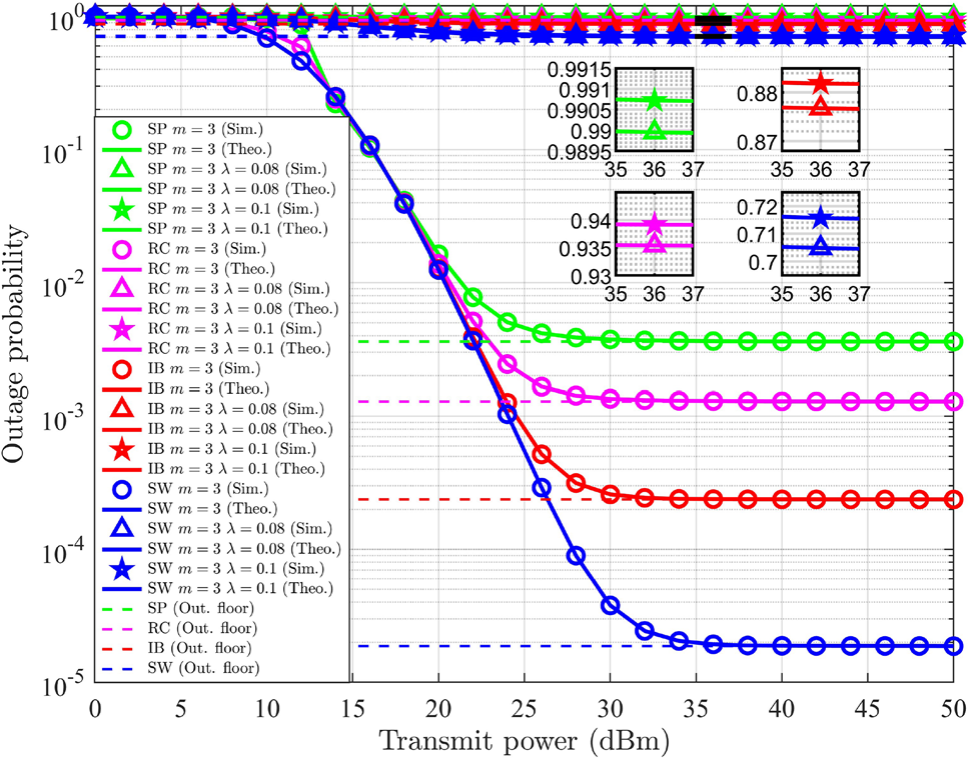
Comparison of average outage probability for $\text {UD}_w$ under various pairing schemes in UL-HNOMA.

**Fig. 19:**
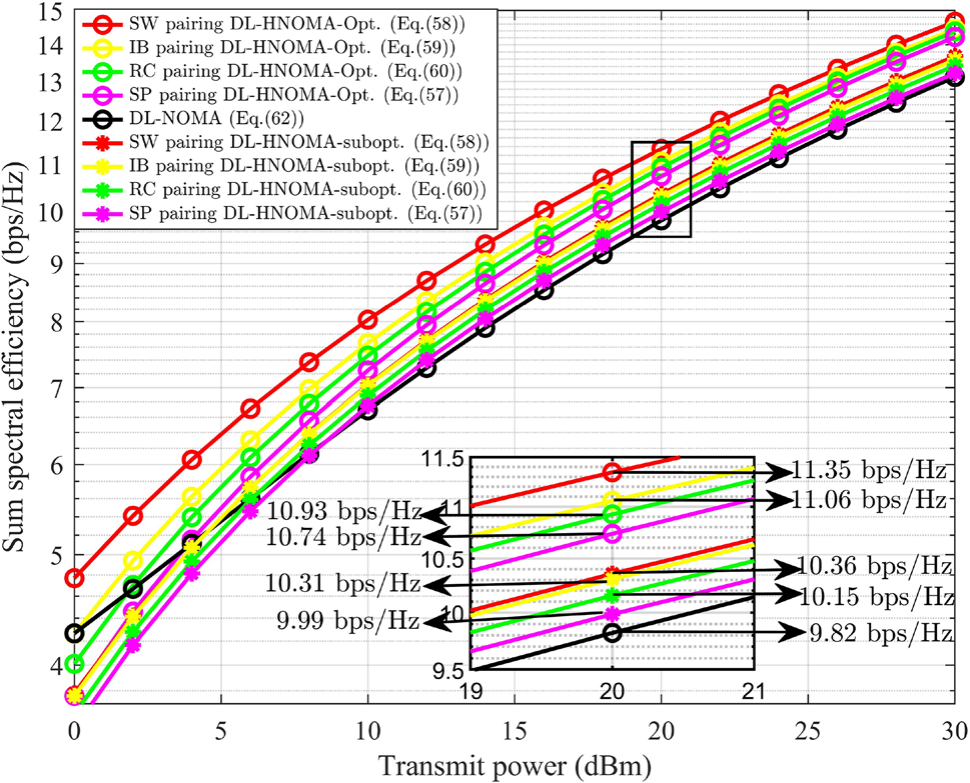
SSE comparison for DL-HNOMA between different pairing schemes with optimal/suboptimal PA factors and DL-NOMA.

**Fig. 20:**
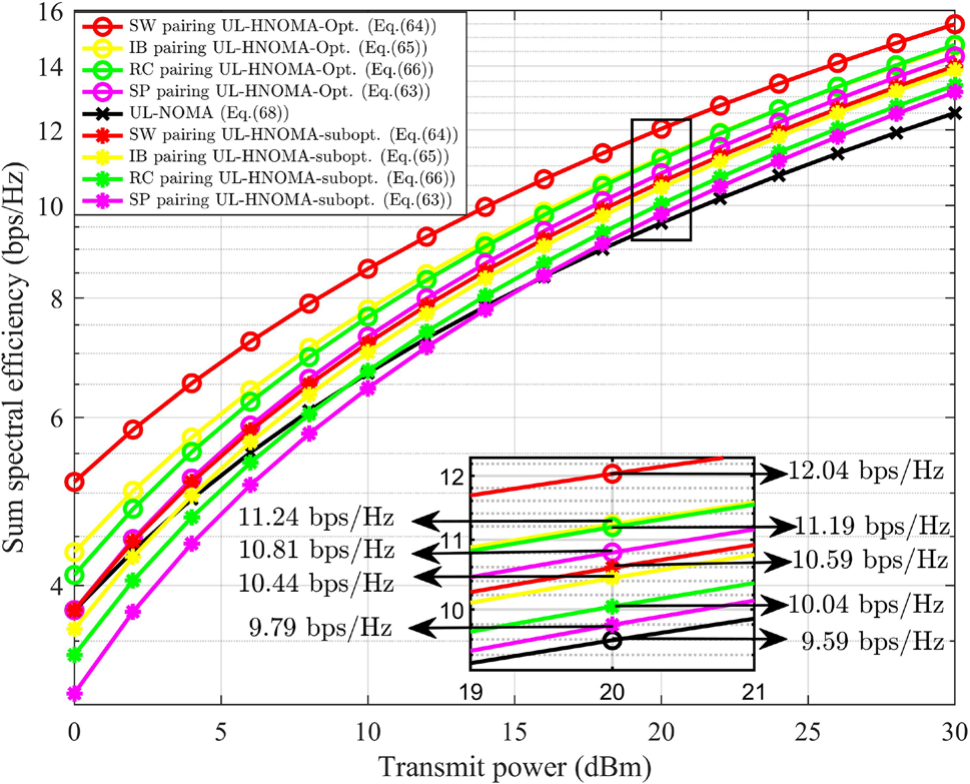
SSE comparison for UL-HNOMA between different pairing schemes with optimal/suboptimal PA factors and UL-NOMA.

The multi-user simulation results are illustrated in Figs. 15, 16, 17, 18, 19, and 20. These results demonstrate that the proposed pairing and PA methods scale effectively beyond two users while maintaining performance gains even under HI and *i*SIC.The simulation results offer a detailed comparison with the traditional NOMA system. This comparison, shown through SSE plots (Figs. 19 and 20), highlights the performance differences between the proposed approach and the baseline approach^17^. To ensure a fair assessment, suboptimal PA factors are used in the simulations^39^. Additionally, user pairing strategies are based on established research, which helps ensure the results are reproducible and credible^7^.

**Table 6:** System parameters for simulation of DL and UL-HNOMA.

Parameter	Values
Nakagami-*m* shape parameter (*m*)	3
Transmit power for DL and UL $(P_{t})$	$-30$ to 50 dBm
Distance of $\text {UD}_1$ from gNB ($\delta _{S-1}$)	300 meters
Distance of $\text {UD}_2$ from gNB ($\delta _{S-2}$)	500 meters
Distance of $\text {UD}_3$ from gNB ($\delta _{S-3}$)	800 meters
Distance of $\text {UD}_4$ from gNB ($\delta _{S-4}$)	1000 meters
Path loss factor ($\eta$)	4
*i*SIC parameter ($\varepsilon$)	0 and 0.1
HI parameter $(\lambda )$	0.08 and 0.1
Number of random channel coefficients (*N*)	$10^7$
Minimum demanded SE for $\text {UD}_1$ ($\tilde{\beta }_1$), $\text {UD}_2$ ($\tilde{\beta }_2$), $\text {UD}_3$ ($\tilde{\beta }_3$), and $\text {UD}_4$ ($\tilde{\beta }_4$)	0.5 bps/Hz

The average probability of outage of the $\text {UD}_s$ for different pairing schemes for DL-HNOMA is depicted in Fig. 15. The graph is explicitly plotted for *m* equal to 3. The impact of HI and *i*SIC is illustrated in the graphs. The values of $\lambda$ considered are 0.08 and 0.1, and $\varepsilon$ is equal to 0.1. The resulting outage increases as $\lambda$ increases for every scheme. Similar to DL-NOMA, for DL-HNOMA, the target outage is set to $10^{-3}$. To achieve the target outage with $\lambda = 0, \varepsilon = 0$, a $P_t$ of $\approx 20$ dBm, $\approx$18.31 dBm, $\approx$11.85 dBm, and $\approx$11.85 dBm is required for SP, RC, IB, and SW pairing, respectively. Table 7 illustrates the $P_t$ in dBm required by various pairing schemes of DL-HNOMA users, $\text {UD}_s$ and $\text {UD}_w$, to achieve an outage probability of $10^{-3}$ for $m = 3$, with varying values of $\lambda$ and $\epsilon$. For $\text {UD}_s$ without HI and *i*SIC, SW and IB pairing outperforms all other schemes. For $\lambda = 0.08, \varepsilon = 0.1$ SW and IB pairing require $\approx$8.17 dBm and $\approx$6.46 dBm less $P_t$ compared to SP and RC schemes. These schemes consider the maximum user separation within a pair, thereby enhancing the outage. The average probability of outage of the $\text {UD}_w$ for different pairing schemes for DL-HNOMA is depicted in Fig. 16. Since each $\text {UD}_w$ directly decodes its own data, its performance is independent of *i*SIC ($\varepsilon = 0$). The considered $\lambda$ values are 0.08 and 0.1, and the resulting outage increases with higher $\lambda$ for every scheme. For $\text {UD}_w$ without HI and *i*SIC, SP pairing outperforms all other schemes. For $\lambda = 0.08, \varepsilon = 0$, SP pairing require $\approx$0.08 dBm, $\approx$0.11 dBm, and $\approx$0.11 dBm less $P_t$ compared to RC, IB and SW schemes. Although SP pairing demonstrates superior performance for the DL-HNOMA $\text {UD}_w$ user, the difference in required transmit power compared to other pairing schemes is minimal.

**Table 7:** $P_t$ in dBm required by various pairing schemes of DL-HNOMA users, $\text {UD}_s$ and $\text {UD}_w$, to achieve an outage probability of $10^{-3}$ for $m = 3$, with varying values of $\lambda$ and $\epsilon$.

Pairing schemes	$\lambda$	Required $P_t$ (dBm) at $\text {UD}_s$ ($\varepsilon = 0$)	Required $P_t$ (dBm) at $\text {UD}_s$ ($\varepsilon = 0.1$)	Required $P_t$ (dBm) at $\text {UD}_w$ ($\varepsilon = 0$)
SP	0	20	–	19.12
	0.08	–	22.47	19.17
	0.1	–	23.13	19.29
RC	0	18.31	–	19.20
	0.08	–	20.76	19.25
	0.1	–	21.43	19.36
IB	0	11.85	–	19.24
	0.08	–	14.30	19.28
	0.1	–	14.97	19.40
SW	0	11.85	–	19.24
	0.08	–	14.30	19.28
	0.1	–	14.97	19.40

The average probability of outage of $\text {UD}_s$ for different pairing schemes in UL-HNOMA is depicted in Fig. 17. Similar to DL-HNOMA, the graph is specifically plotted for *m* equal to 3. The values of $\lambda$ considered are 0.08 and 0.1, and $\varepsilon$ is equal to 0.1. The resulting outage increases as $\lambda$ increases for every scheme. The outage floor comparison of various pairing schemes for UL-HNOMA users, $\text {UD}_s$ and $\text {UD}_w$, is presented for $m = 3$, with varying values of $\lambda$ and $\epsilon$, at a fixed transmit power $P_t$ (in dBm) is presented in Table 8. For $\text {UD}_s$, SW pairing provides the minimum outage floor of $\approx 1.91\times 10^{-5}$ even in the presence of HI ($\lambda =0.08$). The average probability of outage of the $\text {UD}_w$ for different pairing schemes for UL-HNOMA is depicted in Fig. 18. The impact of HI ($\lambda = 0.08, 0.1$) and *i*SIC ($\varepsilon = 0.1$) is illustrated in the graphs. For $\text {UD}_w$, SW pairing provides the minimum outage floor of $\approx 7.05\times 10^{-1}$ even in the presence of HI ($\lambda =0.08$). Compared to $\text {UD}_w$, $\text {UD}_s$ provides the minimum outage floor level with a minimum $P_t$ for every pairing. In UL-HNOMA, SW pairing outperforms all other schemes, which contrasts with DL-NOMA, where the outage of one user is unaffected by the channel of the other. In UL-NOMA, the outage performance of each user is influenced by the channel conditions of different users.

**Table 8:** Outage floor comparison of various pairing schemes for UL-HNOMA users, $\text {UD}_s$ and $\text {UD}_w$, for $m = 3$, with varying values of $\lambda$ and $\epsilon$, at a fixed $P_t$ (in dBm).

Pairing schemes	$\lambda$	Outage floor at $\text {UD}_s$ for $P_t=30$ dBm ($\varepsilon = 0$)	Outage floor at $\text {UD}_w$ for $P_t=34$ dBm ($\varepsilon = 0$)	Outage floor at $\text {UD}_w$ for $P_t=34$ dBm ($\varepsilon = 0.1$)
SP	0	$3.63\times 10^{-3}$	$3.61\times 10^{-3}$	–
	0.08	$3.75\times 10^{-3}$	–	$9.89\times 10^{-1}$
	0.1	$4.07\times 10^{-3}$	–	$9.91\times 10^{-1}$
RC	0	$1.29\times 10^{-3}$	$1.29\times 10^{-3}$	–
	0.08	$1.34\times 10^{-3}$	–	$9.35\times 10^{-1}$
	0.1	$1.45\times 10^{-3}$	–	$9.39\times 10^{-1}$
IB	0	$2.35\times 10^{-4}$	$2.37\times 10^{-4}$	–
	0.08	$2.43\times 10^{-4}$	–	$8.76\times 10^{-1}$
	0.1	$2.66\times 10^{-4}$	–	$8.82\times 10^{-1}$
SW	0	$1.84\times 10^{-5}$	$1.88\times 10^{-5}$	–
	0.08	$1.91\times 10^{-5}$	–	$7.05\times 10^{-1}$
	0.1	$2.09\times 10^{-5}$	–	$7.16\times 10^{-1}$

The SSE comparison for DL-HNOMA for different pairing schemes with optimized PA factors and DL-NOMA is illustrated in Fig. 19. The graph is specifically plotted for *m* equal to 3 and a number of users of $M=4$. The optimal and suboptimal values for PA factors are included using (72) and (73), respectively. The SSE expressed with optimal PA factors is labeled as DL-HNOMA-opt, while those with suboptimal PA factors are labeled as DL-HNOMA-subopt. The DL-NOMA system without pairing and PA optimization is denoted as DL-NOMA. In the case of DL-NOMA, fixed PA is considered. The $\text {UD}_4$, being the farthest from the gNB, is allocated a fixed PA of 0.6. Following this, $\text {UD}_3$ is assigned three-fourths of the remaining $P_t$. Next, $\text {UD}_2$ receives three-fourths of what is left. Finally, $\text {UD}_1$ is given the remaining $P_t$. Hence, the PA factors considered for $\text {UD}_4$, $\text {UD}_3$, $\text {UD}_2$, and $\text {UD}_1$ are 0.6, 0.3, 0.075, and 0.025. The SSE for different pairing schemes and DL-NOMA is performed using (57), (58), (59), (60), and (62), respectively. To compare the analysis, a $P_t$ of 20 dBm is considered. The SSE achieved for SW pairing DL-HNOMA-opt is $\approx$11.35 bps/Hz, and for SW pairing DL-HNOMA-subopt is $\approx$10.36 bps/Hz. The SSE obtained for IB pairing DL-HNOMA-opt is $\approx$11.06 bps/Hz, and for IB pairing DL-HNOMA-subopt is $\approx$10.31 bps/Hz. The SSE obtained for RC pairing DL-HNOMA-opt is $\approx$10.93 bps/Hz, and for RC pairing DL-HNOMA-subopt is $\approx$10.15 bps/Hz. For SP pairing DL-HNOMA-opt, the SSE obtained is $\approx$10.74 bps/Hz, while SP pairing DL-HNOMA-subopt achieves the SSE of $\approx$9.99 bps/Hz. The SSE attained by DL-NOMA for the corresponding $P_t$ is $\approx$9.82 bps/Hz. The findings indicate that the SW pairing DL-HNOMA-opt scheme outperforms all other methods, achieving a maximum improvement in SSE of $\approx$15.58%. The performance of SW pairing DL-HNOMA-opt is primarily attributable to maximizing the spatial separation between the users in each pair, alongside the incorporation of optimized PA factors. This strategy effectively enhances the SSE across diverse channel conditions. The SSE comparison of various pairing schemes in both DL and UL-HNOMA is presented in Table 9 for both optimal and suboptimal PA strategies.

The SSE comparison for UL-HNOMA for different pairing schemes with optimized PA factors and UL-HNOMA is illustrated in Fig. 20. The optimal and suboptimal values for PA factors are included using (75) and (76), respectively. The plot used similar parameters and notations as in DL-HNOMA. The PA factors considered for $\text {UD}_1$, $\text {UD}_2$, $\text {UD}_3$, and $\text {UD}_4$ are 0.6, 0.3, 0.075, and 0.025. The SSE for different pairing schemes and DL-NOMA is performed using (63), (64), (65), (66), and (68), repectively. To compare the analysis, a $P_t$ of 20 dBm is considered. The findings indicate that, similar to DL-HNOMA, the SW pairing UL-HNOMA-opt scheme outperforms all other methods, achieving a maximum improvement in SSE of $\approx$25.55%. The analysis was conducted with *m* equal to 3. However, increasing the value of *m* can achieve additional improvements.

**Table 9:** Comparison of SSE for different pairing schemes in both DL- and UL-HNOMA under optimal and suboptimal PA strategies.

Schemes	DL-HNOMA		UL-HNOMA	
	SSE (bps/Hz)	% improvement of SW pairing over other schemes	SSE (bps/Hz)	% improvement of SW pairing over other schemes
SW pairing-opt	11.35	–	12.04	–
IB pairing-opt	11.06	2.62%	11.24	7.12%
RC pairing-opt	10.93	3.84%	11.19	7.59%
SP pairing-opt	10.74	5.68%	10.81	11.38%
SW pairing-subopt	10.36	9.56%	10.59	13.69%
IB pairing-subopt	10.31	10.09%	10.44	15.33%
RC pairing-subopt	10.15	11.82%	10.04	19.92%
SP pairing-subopt	9.99	13.60%	9.79	22.98%
NOMA	9.82	15.58%	9.59	25.55%

The proposed scheme enhances SE and SSE, offering significant benefits for real-time applications in future wireless systems. As the radio spectrum is a valuable and limited resource, enhancing SE enables the transmission of more data using the same bandwidth. It is crucial for accommodating the massive number of devices and services expected in 6G networks and the IoT. This improvement helps the network providers to reduce deployment expenses while enhancing overall capacity. Users experience a higher data rate and reduced latency. It also facilitates activities such as high-definition streaming, augmented reality, virtual reality, and autonomous technologies. Additionally, higher SE with less transmission power provides an energy-efficient and sustainable network. Significantly, our findings indicate that these improvements in SE are valid even in realistic scenarios, including Nakagami-*m* fading, HI, and *i*SIC, highlighting the practical reliability of our method.

## Conclusion

This investigation provides a detailed outage analysis for both DL and UL-NOMA over Nakagami-*m* fading channels, deriving closed-form solutions for outage probability under HI and *i*SIC. The study highlights the significant impact of the LOS component (*m*) on outage performance. Results show that increasing *m* drastically reduces the required transmit power $(P_t)$ for a given outage threshold in DL-NOMA (e.g., up to $\approx 23.93$ dBm reduction for $m=1$ vs. $m=8$) and significantly lowers the outage floor in UL-NOMA (e.g., for $\text {UD}_1$, from $\approx 2\times 10^{-2}$ to $\approx 2.32\times 10^{-9}$ when *m* increases from 1 to 8). The analysis also quantifies the detrimental effects of HI and *i*SIC on outage performance, showing increased $P_t$ or outage floor with higher impairment levels in both DL and UL.

Furthermore, the study evaluates various user pairing strategies. In DL-HNOMA, SW and IB pairing schemes achieve the minimum average outage probability for strong users, with SW pairing demonstrating superior performance overall, requiring up to $\approx 8.15$ dBm lower $P_t$ compared to other schemes. For weak users, SP provides minimal outage. In UL-HNOMA, SW pairing consistently yields the lowest outage floor for both strong $(\approx 1.91\times 10^{-5})$ and weak $(\approx 7.05\times 10^{-1})$ users under HI conditions, significantly outperforming other schemes like RC, IB, and SP pairing which show higher outage floors. The analysis states that in UL, a user’s outage depends on the channel gains of other users, resulting in different average outage conditions compared to DL.

Finally, the research demonstrates the effectiveness of SW pairing with optimal PA factors in maximizing SSE. SW pairing achieves an SSE of $\approx 11.35$ bps/Hz in DL-HNOMA and $\approx 12.04$ bps/Hz in UL-HNOMA, representing a maximum improvement of $\approx 15.58\%$ in DL and $\approx 25.55\%$ in UL compared to other pairing methods. The findings underscore the importance of LOS components, impairment mitigation, and optimized user pairing for enhancing NOMA system performance. The proposed scheme demonstrates significant enhancements in SSE and remains robust even when facing Nakagami-m fading, HI, and *i*SIC. However, there are some limitations to consider. For example, the Nakagami-*m* channel model becomes inaccurate and unrealistic for $m < 0.5$, indicating extremely severe fading. The current PA and pairing methods rely on precise channel information, but in real-time scenarios, errors in channel estimation can affect performance. Future research will aim to expand the analysis to larger user groups with higher mobility. The goal is to develop more robust PA strategies that are effective even with imperfect channel information, while achieving multiple performance objectives. Future work will explore more generalized fading models, RIS technology integration, and artificial intelligence (AI)-driven power optimization.

## Supplementary Information

Supplementary Information.

## Data Availability

The datasets used and/or analyzed during the current study are available from the corresponding author on reasonable request.
