# Supplementary material for: Spectral efficiency enhancement in multiuser NOMA over Nakagami-m fading channels through power allocation and pairing strategies under hardware and SIC imperfections
Source: Sci Rep. 2025 Oct 31;15:38211. doi: 10.1038/s41598-025-22024-z (PMC12578920; doi:10.1038/s41598-025-22024-z)
Supplement: Supplementary file 1 — Supplementary Information. [file 41598_2025_22024_MOESM1_ESM.pdf]

Supplementary Material

The detailed derivations of equations (71) and (74) are provided in Appendices A and B, respectively.

A Derivation for optimal PA in DL-NOMA

The outage condition for UD_1 is denoted as $(R_{s-1}^{DL} < \tilde{\beta}_1) \cup (R_{UD_1}^{UD_2} < \tilde{\beta}_2)$. Hence, the condition without outage can be represented as, $(R_{s-1}^{DL} \geq \tilde{\beta}_1) \cup (R_{UD_1}^{UD_2} \geq \tilde{\beta}_2)$. Consider the first condition $(R_{s-1}^{DL} \geq \tilde{\beta}_1)$, and substitute R_{s-1}^{DL} in the condition using (6), which results in,

$$\log_2(1 + g_{s-1}\alpha_1\rho_n) \geq \tilde{\beta}_1 \tag{a}$$

Substituting $\beta_1 = 2^{\tilde{\beta}_1} - 1$, and rearranging the equation result in an expression for the minimum value for α_1 to provide the fairness, and it is expressed as,

$$\alpha_1 \geq \frac{\beta_1}{g_{s-1}\rho_n} \tag{b}$$

Consider the second condition $(R_{UD_1}^{UD_2} \geq \tilde{\beta}_2)$, and substitute $R_{UD_1}^{UD_2}$ in the condition using (5), which results in,

$$\log_2\left(1 + \frac{g_{s-1}\alpha_2\rho_n}{g_{s-1}\alpha_1\rho_n + 1}\right) \geq \tilde{\beta}_2 \tag{c}$$

Substituting $\beta_2 = 2^{\tilde{\beta}_2} - 1$, and $\alpha_2 = 1 - \alpha_1$ and rearranging the equation result in an expression for the maximum value for α_1 to provide fairness, and it is expressed as,

$$\alpha_1 \leq \frac{g_{s-1}\rho_n - \beta_2}{g_{s-1}\rho_n(\beta_2 + 1)} \tag{d}$$

By combining the conditions of (b) and (d), the optimal range for PA factors for maximum SSE with minimum outage for DL-NOMA will be obtained as in (71). The corresponding value for α_2 will be obtained using the expression $\alpha_2 = 1 - \alpha_1$. Using (71), the optimal and suboptimal value for α_1 in DL-NOMA is identified.

B Derivation for optimal PA in UL-NOMA

The outage condition for UL-NOMA is denoted as $(R_{s-1}^{UL} < \tilde{\beta}_1)$ and $(R_{s-2}^{UL} < \tilde{\beta}_2)$. Hence, the condition without outage can be represented as, $(R_{s-1}^{UL} \geq \tilde{\beta}_1)$ and $(R_{s-2}^{UL} \geq \tilde{\beta}_2)$, which provides a minimum fairness guarantee to both users. By substituting R_{s-1}^{UL} in the first condition using (13), which results in,

$$\log_2\left(1 + \frac{g_{s-1}\alpha_1\rho_n}{g_{s-2}\alpha_2\rho_n + 1}\right) \geq \tilde{\beta}_1 \tag{e}$$

Substituting $\beta_1 = 2^{\tilde{\beta}_1} - 1$, and $\alpha_2 = 1 - \alpha_1$ and rearranging the equation result in,

$$\alpha_1 \geq \frac{\beta_1(1 + g_{s-2}\rho_n)}{(g_{s-1} + g_{s-2}\beta_1)\rho_n} \tag{f}$$

By substituting R_{s-2}^{UL} in the second condition using (13), which results in,

$$\log_2(1 + g_{s-2}\alpha_2\rho_n) \geq \tilde{\beta}_2 \tag{g}$$

Substituting $\beta_2 = 2^{\tilde{\beta}_2} - 1$, and $\alpha_2 = 1 - \alpha_1$ and rearranging the equation result in,

$$\alpha_1 \leq \frac{g_{s-2}\rho_n - \beta_2}{g_{s-2}\rho_n} \tag{h}$$

By combining the conditions of (f) and (h), the optimal range for PA factors for maximum SSE with minimum outage for UL-NOMA will be obtained as in (74). The corresponding value for α_2 will be obtained using the expression $\alpha_2 = 1 - \alpha_1$. Using (74), the optimal and suboptimal value for α_1 in UL-NOMA is identified.
